# Lower extremity robotic exoskeleton devices for overground ambulation recovery in acquired brain injury—A review

**DOI:** 10.3389/fnbot.2023.1014616

**Published:** 2023-05-25

**Authors:** Kiran K. Karunakaran, Sai D. Pamula, Caitlyn P. Bach, Eliana Legelen, Soha Saleh, Karen J. Nolan

**Affiliations:** ^1^Center for Mobility and Rehabilitation Engineering Research, Kessler Foundation, West Orange, NJ, United States; ^2^Department of Physical Medicine and Rehabilitation, Rutgers—New Jersey Medical School, Newark, NJ, United States; ^3^Research Staff Children's Specialized Hospital New Brunswick, New Brunswick, NJ, United States; ^4^Department of Psychology, Montclair State University, Montclair, NJ, United States

**Keywords:** cerebral palsy, traumatic brain injury, robotic exoskeleton, gait, balance, rehabilitation, stroke

## Abstract

Acquired brain injury (ABI) is a leading cause of ambulation deficits in the United States every year. ABI (stroke, traumatic brain injury and cerebral palsy) results in ambulation deficits with residual gait and balance deviations persisting even after 1 year. Current research is focused on evaluating the effect of robotic exoskeleton devices (RD) for overground gait and balance training. In order to understand the device effectiveness on neuroplasticity, it is important to understand RD effectiveness in the context of both downstream (functional, biomechanical and physiological) and upstream (cortical) metrics. The review identifies gaps in research areas and suggests recommendations for future research. We carefully delineate between the preliminary studies and randomized clinical trials in the interpretation of existing evidence. We present a comprehensive review of the clinical and pre-clinical research that evaluated therapeutic effects of RDs using various domains, diagnosis and stage of recovery.

## 1. Introduction

Acquired brain injury (ABI) is a leading cause of ambulation deficits affecting people in the United States every year (Centers for Disease Control and Prevention, 2016; Menon and Bryant, 2019; CDC Stroke Statistics, 2020; Alliance BI, 2023). An ABI is an injury to the brain that is not hereditary, or degenerative (Menon and Bryant, 2019; BIA, 2021). The injury results in a change to the brain's neuronal activity, which may affect the physical integrity, metabolic activity, or functional ability of nerve cells in the brain, and in turn affects function (BIA, 2021). For the purpose of this review ABI refers to a diagnosis of stroke, traumatic brain injury, and cerebral palsy.

Sixty-five percent of individuals diagnosed with ABI (stroke, traumatic brain injury, and cerebral palsy) have mobility deficits; despite rehabilitation, over half of them present with functional ambulation deficits even after 1 year, limiting their community ambulation, independence, and activities of daily living (ADL) (Wade and Hewer, 1987; Friedman, 1990). Regaining ambulation is a priority in adults and children with ABI to improve their participation and quality of life (QOL) (Rudberg et al., 2021).

Post-ABI gait and balance rehabilitation is based on the theory that consistent, repeated task-specific practice will lead to recovery of function (Partridge et al., 2000; Cooke et al., 2010). Some of the critical parameters for improving mobility post-ABI are task-specific, repetitive practices that are progressively more challenging (Langhorne et al., 2009). Wearable robotic devices for over-ground walking offer an alternative modality for rehabilitation, because they can facilitate task-specific, repetitive practice that is progressively more challenging for individuals with acute and chronic ABI.

The past decade has witnessed a dramatic growth in the study and application of wearable robotic devices (RDs) for over-ground gait training in individuals with ABI (Canela et al., 2013; Murray et al., 2015; Federici et al., 2016; Louie and Eng, 2016; Kozlowski et al., 2017; Lefeber et al., 2017; Lerner et al., 2017a; Patané et al., 2017; Molteni et al., 2018; Androwis et al., 2019; Karunakaran et al., 2019; Moucheboeuf et al., 2020). RDs can provide trajectory guidance and assistance at various joints individually (hip, knee, ankle) or in combination (multi-joint), to assist, resist, or augment muscle torque (Dollar and Herr, 2008; Yan et al., 2015; Esquenazi et al., 2017; Iandolo et al., 2019). Some RDs also provide rigid support for stability and static balance (Ekso, ReWalk, HAL, etc.) to keep the users in an upright posture (Dollar and Herr, 2008; Yan et al., 2015; Esquenazi et al., 2017; Iandolo et al., 2019). These exoskeletons have a rigid structure at the joints and/or links. They may provide hip or/and back support to keep the users in the upright position. Though these robots do not provide dynamic balance control. This upright posture is very important during gait training to provide quality repetitions especially in people who require maximum assistance from therapists. Therapy requirements differ based on time since injury and deficits (Kwakkel et al., 2004; Neural Plasticity After Acquired Brain Injury, 2022). It is well established that recovery plateaus with time, which is why repetitive practice should start as early as possible to change the trajectory of recovery (Kwakkel et al., 2004; Neural Plasticity After Acquired Brain Injury, 2022). RDs are capable of early mobilization, providing consistent repetitive physical therapy by assisting users with severe gait and balance deficits early after ABI. With time (i.e., in chronic stages of ABI), many patients develop compensatory mechanisms such as circumduction, steppage gait, hip hiking, toe walking, to successfully ambulate (Kerrigan et al., 2000; Williams et al., 2009; Winter, 2009; Kemu, 2010; Perry and Burnfield, 2010; Dubin, 2014; Sheffler and Chae, 2015). These pathological deviations from healthy walking result in slower walking speed, shorter step length, decreased symmetry, reduced gait and balance adaptability, and increased risk of falls (Kerrigan et al., 2000; Williams et al., 2009; Winter, 2009; Kemu, 2010; Perry and Burnfield, 2010; Dubin, 2014; Sheffler and Chae, 2015). One of the goals of therapy is to reduce these compensatory mechanisms and train individuals to perform healthy and efficient overground ambulation. RDs are functionally capable of providing this training and can be useful in both the acute and chronic stages of recovery (Calabrò et al., 2018; Molteni et al., 2018, 2021; Nolan et al., 2020; Rojek et al., 2020).

Despite rapid progress in robotic exoskeleton design and technology, limited data is available on the evaluation of RD efficacy with regard to children and adults diagnosed with ABI. In order to fully understand the effects of RDs, it is imperative to answer questions related to their utilization, such as: (a) How does early RD therapy change the recovery curve?; (b) Who should use multi-joint RD vs. single joint RD?; (c) Does a person with low deficits benefit from multi-joint or single joint RD therapy during the acute stages of recovery?; (d) Would it be beneficial to use a single joint robot over multi-joint robot to target a deficit or reduce a compensatory mechanism?; and (e) How does providing assistance/resistance change the way we learn? In order to answer these questions, we need to understand: (1) the effect of RDs on functional recovery, as well as biomechanical, physiological, and cortical mechanisms, and (2) the effect of mechanical and software (control) characteristics of RDs on time since injury, as well as on the various deficits. To date, most published studies have analyzed functional recovery, but there is limited research on the effect of RD over-ground gait training on biomechanical, physiological, and cortical mechanisms in children and adults with ABI. Biomechanical and physiological outcomes quantitatively reflect the underlying impairment in joint mechanisms, inter-limb coordination or balance mechanisms, and their recovery. Understanding the changes in gait and balance mechanisms will help us understand the reasons for the observed functional changes and will help us to better understand recovery. Research on structural and functional changes in the cortical and subcortical levels will help us understand the underlying mechanisms of neuroplasticity. Therefore, comprehensive efficacy studies across parameters will help us understand the effects of RDs and how to improve rehabilitation strategies. Most of the available literature reviews on exoskeleton research have focused on design and development activities in terms of electromechanical design or software controllers to provide optimal and efficient device (Dollar and Herr, 2008; Viteckova et al., 2013; Shi et al., 2019; Lee et al., 2020). Other reviews were on gait trainers/non-overground robotic devices that are very different from overground robotic exoskeletons (Moucheboeuf et al., 2020). Several reviews had a narrower focus; such as reviews on only randomized clinical trials or reviews of safety, ease of use, or feasibility of use in clinical environments (Mehrholz and Pohl, 2012; Poli et al., 2013; Federici et al., 2015, 2016; Schwartz and Meiner, 2015; Wall et al., 2015; Louie and Eng, 2016; Alias et al., 2017; Hill et al., 2017; Jayaraman et al., 2017; Lefeber et al., 2017; Bruni et al., 2018; Mehrholz et al., 2018; Molteni et al., 2018; Weber and Stein, 2018; Postol et al., 2019; Moucheboeuf et al., 2020; Pinto-Fernandez et al., 2020; Swank et al., 2020a; Dijkers et al., 2021; Sale et al., 2021). Though they provide a great insight into the usage of the device, they do not help us to understand the relationship between training, neuroplasticity, and functional recovery.

This review is targeted at researchers and developers in the field of robotic neurorehabilitation. The goal is to provide a comprehensive review of state of the science, i.e., the clinical and pre-clinical research on the therapeutic effects of various over-ground gait training RDs, and to identify gaps in research areas in order to identify directions for further investigation. We present and discuss existing assessments in terms of functional, clinical, biomechanical, physiological, and cortical mechanisms. We also provide guidelines and recommendations for clinical and pre-clinical research, taking into account the clinical needs of the patient population.

## 2. Methodology

This review was conducted in accordance with the framework proposed by Moher et al. (2009). PubMed, and Scopus databases were accessed and searched from inception to July 31, 2021. We combined the search terms (lower extremity exoskeletons OR lower limb exoskeleton OR gait exoskeleton OR exoskeleton ambulation OR exoskeleton walking), with humans and English language as limits. All duplicates between the search criteria were removed.

Inclusion criteria were full-text, peer-reviewed articles that used a powered robotic exoskeleton device (RD) with adults and children post acquired brain injury as an intervention for overground gait rehabilitation. Articles were included if they reported functional outcomes (e.g., speed, distance, independence, etc.), clinical outcomes [e.g., Functional Independence Measure (FIM), Gross Motor Function Classification (GMFC), etc.], biomechanical outcomes (e.g., kinematic, kinetic, temporal-spatial, etc.), physiological [e.g., Electromyography (EMG), etc.] and neurological [e.g., Magnetic Resonance Imaging (MRI), functional Magnetic Resonance Imaging (fMRI), functional Near Infrared Spectroscopy (fNIRS), etc.].

Lower extremity RDs are herein operationally defined as a wearable robotic device that actuates at least one of the three lower extremity joints (hip, knee, and ankle) during overground gait either unilaterally or bilaterally in one or more movement planes'. Articles were excluded if they were on neurological conditions other than ABI; articles on industrial and military applications; reported only technology development; reported only orthotic effect of RD; reported only feasibility of usage; included only healthy participants; utilized a treadmill-based device; or if only an abstract was available. Titles and abstracts were screened for relevance by two authors according to the inclusion and exclusion criteria above. In the event of conflict, a third author was consulted for resolution. Full-texts were then screened, and reference lists of all selected articles were searched for additional studies. Included articles were then examined to extract data regarding study design, RD, participant characteristics, intervention, training period, outcome measures, adverse effects, and results. We examined the changes in functional, clinical, biomechanical, physiological, and neurological outcomes published in the qualifying literature.

**Figure 1:**
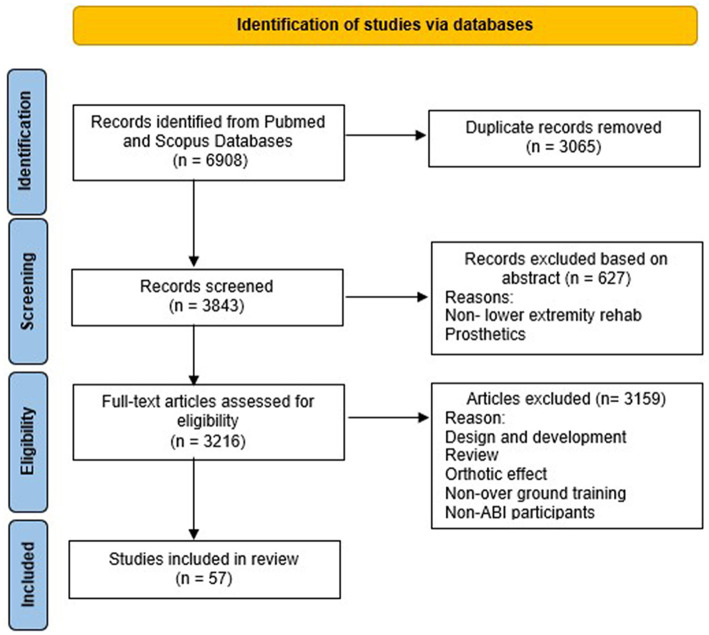
Flow diagram of studies identification.

A total of 6,908 articles were retrieved using the search criteria. After removing the articles based on the inclusion and exclusion criteria (Figure 1), 57 articles remained and were included for this review.

## 3. Results

**Figure 2:**
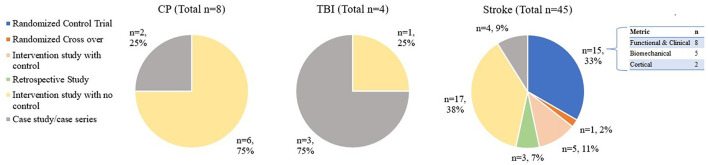
Studies divided based on population and further divided based on type of study. Some of the studies with Biomechanical and cortical outcomes also presented functional and studies with functional outcome also presented biomechanical metrics as secondary outcomes. Please refer to Table 1 for all the outcomes measures.

**Figure 3:**
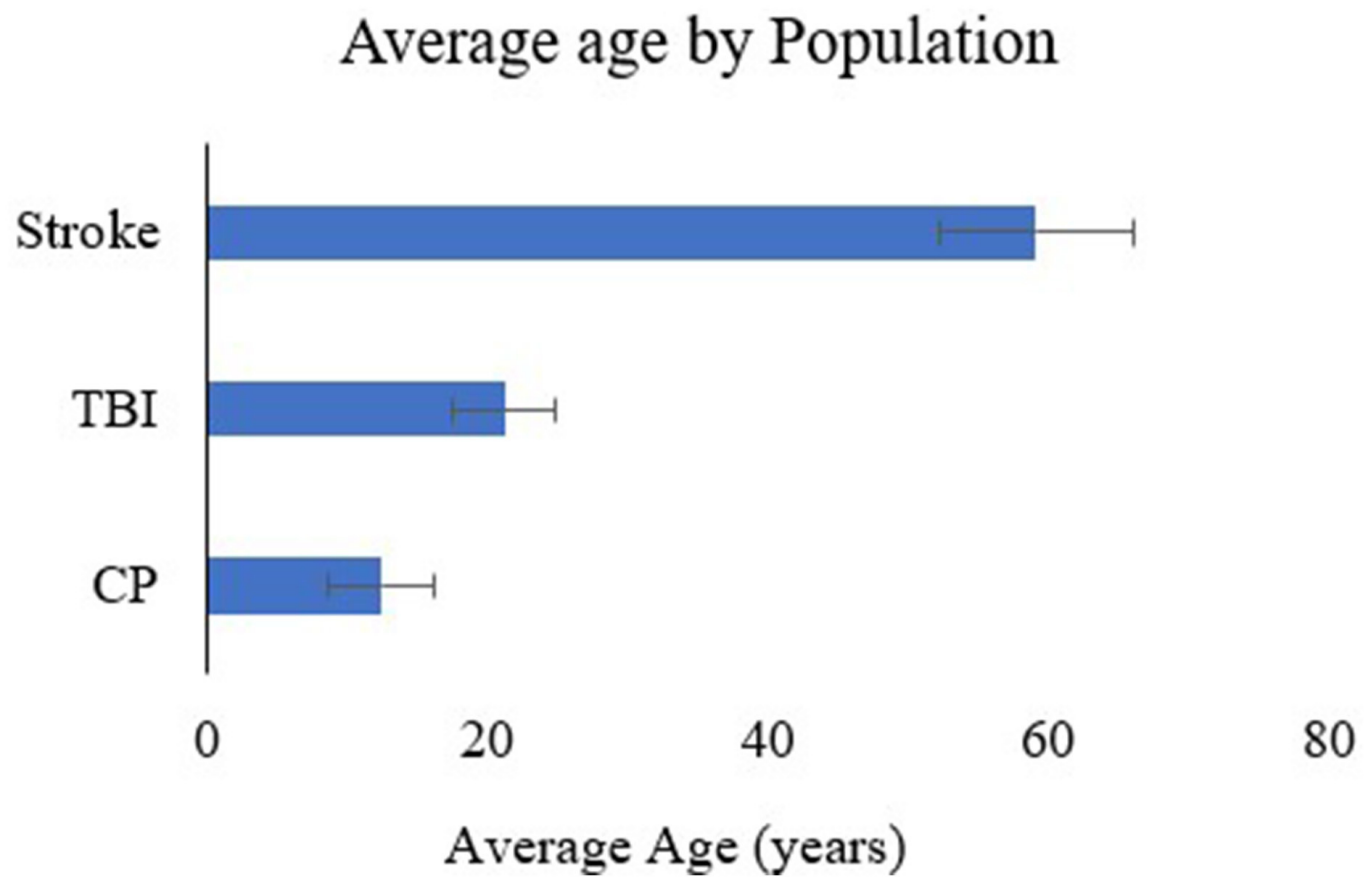
Average age by population.

Figure 2 shows all the studies included in this review to give an overview of the state of the science in the field of therapeutic exoskeletons. RD Research is sparce in TBI and CP diagnosis, with no randomized clinical trials (RCT's) or intervention studies with a control group currently available in these two populations. In stroke, about half the studies have a control group (i.e., randomized control trial, randomized cross over, intervention study with control and retrospective study). Figure 3 shows the average age by population. Studies on CP (6 studies < 18 years; 2 studies < 31 years) and TBI (ages >13 and < 30 years) have predominantly been on pediatric and young adults. On the other hand, all stroke studies have been on middle age to older adults (ages >35 and < 80 years). Studies on CP had participants with quadriplegia (1 study), and diplegia (7 studies), while all participants in TBI and stroke had one sided weakness (hemiplegia). Age, affected side, sex, and diagnoses are detailed in Table 1.

**Table 1:** Pre-clinical and clinical research on exoskeletons in CP, TBI and stroke.

**References**	**Study type**	**Research objectives**	**Demographics**	**Intervention**	**Evaluation**	**Findings**
**Cerebral palsy (CP): Biomechanical and physiological outcomes**						
Kuroda et al. (2020)	Intervention case study with pre-post evaluation	Examine the effect of 2s-HAL RD improvement in functional and clinical gait outcomes for pediatric CP	Chronic quadriplegic CP: *n* = 1 Age: 11	HAL RD, 12 sessions, 40–60 min/session for 4 weeks	Timeframe: BL-post, 1 month, 2-month, and 3-month post Functional: GS, 10MWT, 6MWT Clinical: PCI, GMFCS, COPM Biomechanical: SL and CAD	BL to post: GS, CAD, SL, 6MWT, GMFCS and COPM ↑, PCI ↓ SL peaked at 1 month, GS peaked at 2 months, CAD peaked at 3 months No significance calculated
Bayón et al. (2016)	Intervention study, pre-post evaluation with no control group	Evaluate CPWalker RD improvements in biomechanical outcomes for CP	Chronic spastic diplegic CP *n* = 3 Age: 11–18	CPWalker RD, 10 sessions, 1 h for 5 weeks	Timeframe: BL-Post Biomechanical: Velocity, CAD, SL	BL to post: Velocity↑, CAD ↑, SL ↑ No significance calculated
Bayón et al. (2018)	Intervention study, pre-post evaluation with no control group	Evaluate robotic rehabilitation therapy for CP using the CPWalker	Diplegic CP *n* = 4 Age: 12–17	CPWalker RD, 16 sessions, 1 h for 8 weeks	Timeframe: BL-Post Functional: 10MWT, 6MWT Clinical: PCI, Selective Control Assessment of Lower Extremity (SCALE), GMFCS Biomechanical: Isometric Strength, range of motion	BL to post: MCID reached for two patients in 10MWT and 6MWT, all patients ↓ PCI, all patients ↑ SCALE, ↑Isometric Strength, and ↑ range of motion. No significance calculated
Lerner et al. (2017a)	Intervention case study with pre-post evaluation	Evaluate if novel RD motorized knee extension improves biomechanical and physiological outcomes in CP	Subacute diplegic CP *n* = 1 Age: 6	Novel RD, 5 sessions	Timeframe: BL-Post Conditions: with/without motor Biomechanical: HA, KA, AA, CAD, SL, SW Physiological: EMG Of RF, VL, SEMI-T, MG	BL to post in free: max AA^*^, CAD^*^ BL to post in assistive: AA^*^, stance KF^*^and KE^*^, knee range of motion^*^, CAD^*^ No motor to with motor: SEMI-T^**^ in favor of motor condition
Lerner et al. (2017b)	Intervention study, pre-post evaluation with no control group	Evaluate Novel RD for treatment of flexed knee gait for children with CP	Diplegic CP *n* = 7 Age: 5–19	Novel RD, 6 sessions, 2–3 h each	Timeframe: BL-post Conditions: RD with stance, swing, and with both stance-swing assist Biomechanical: KA, SL, CAD, GS Physiological: EMG activity of VL, SEMI-T	BL to post: KE in midstance^*^ at initial contact and in stance and swing assist condition^*^, VL activity^*^ and SEMI-T activity^*^ during stance, swing, and both conditions
Bulea et al. (2018)	Intervention study, pre-post evaluation with no control group	Determine if Novel RD can improve variance ratio of VL and SEMI-T muscles during gait for children with CP	Chronic diplegic *n* = 7 Age: 5–20	Novel RD, 6 sessions, 2–3 h each	Timeframe: BL-Post Conditions: KE assistance in stance, late-swing, or both Physiological: Variance ratio of VL, and SEMI-T from EMG	BL to post: Variance ratio of VL^*^ and SEMI-T^*^
Fang et al. (2020)	Intervention study, pre-post evaluation with no control group	Evaluate the effects of personalized ankle plantar and dorsi flexor assistance Biomechanical and physiological outcomes CP	Diplegic CP *n* = 6 Age: 9–31	Ankle RD, 4 sessions of 2–10-minute bouts of walking around a 61-m oval track	Timeframe: BL-Post for CGT and RD walking, post CGT to post RD, BL-CGT in first session to post RD in final session Biomechanical: GS, SLL, CAD Physiological: EMG activity in SEMI-T, SO and VL	BL to post for CGT: GS^*^, SLL^*^, variability in SO^*^ and VL^*^ BL to post for RD: GS^*^, SLL^*^, variability in SO^*^ and VL^*^ Post CGT to post RD: GS^*^ and SLL^*^ in RD BL CGT to post RD: GS^*^ and SLL^*^
**Traumatic brain injury (TBI): Biomechanical and physiological outcomes**						
Ekso RD gait training on biomechanical outcomes	Acute TBI right sided weakness *n* = 1 Age: 21	Ekso RD, 12 sessions, 30 min/session	Timeframe: BL-Post Biomechanical: KA, HA, AA, SL, lateral foot displacement, GS, total time, swing and stance time of affected side	BL to post: ↓ joint angle variability swing, SL and GS, ↑ in stance and Lateral Foot Displacement, No significance calculated
Karunakaran et al. (2019)	Intervention case study with pre-post evaluation	Evaluate the effect of Ekso RD gait training on biomechanical outcomes	Chronic TBI *n* = 1, Healthy Control (HC) *n* = 1	Ekso RD, 12 sessions, 50 min/day	Timeframe: BL-Post Biomechanical: Total normal force (TNF), spatial-temporal symmetry, SL, GS, STT, SWT	BL to post: TNF profile similar to HC at follow-up, ↑ spatial symmetry, STT, SWT, SL, GS
Karunakaran et al. (2020a)	Intervention study, pre-post evaluation with no control group	Evaluate RD training effect on loading/unloading and spatial characteristics for chronic ABI	Chronic TBI: *n* = 4 stroke: *n* = 2 HC *n* = 1 6L/1R Age: 14–27	Ekso RD, 12 sessions, 45 min each HC one session without RD used for reference	Timeframe: BL-Post Biomechanical: Total vertical pressure, linearity of loading (LOL), rate of LOL, GS, SL, average total time, STT, SWT, DST	BL to post: ↑Total vertical pressure, LOL^*^, ↑ in SL, speed, and GS, ↓ in total, stance, and DST with no significant effect
**Traumatic brain injury (TBI): Neurological outcomes**						
Karunakaran et al. (2020b)	Intervention case study with pre-post evaluation	Examine cortical outcome in chronic TBI patients after Ekso RD intervention using fNIRS	Chronic TBI Right sided weakness: *n* = 1 HC: *n* = 1 Age: 22, 26	Ekso RD, 50 min/day12 sessions, 6 blocks of 20 s rest and 20 s	Timeframe: BL-Post Condition: walking with and w/o RD HC participated w/o RD Cortical: fNIRS Functional: GS, 10MWT, 2MWT, TUG	BL to post: ↓ activation shown in prefrontal cortex, motor cortex, and bilateral pre-motor cortex, ↑ in speed, TUG, 2MWT. No significance was calculated
**Stroke- Rigid exoskeletons: Functional and clinical outcomes**						
Karunakaran et al. (2021)	Interventional study comparing pre-post effects with no control group	Evaluate the differences between Ekso GT RD training and CGT on functional gait outcomes in stroke	Acute stroke: *n* = 14 10L/4R Age: 18–82	Ekso GT, RD+CGT during PT session, 45 min to 90 min/session	Timeframe: BL-post Conditions: RD + CGT Functional: WD, total steps, steps per session, 10MWT, 6MWT, TUG	BL to post: TUG^*^, 10MWT^*^, 6MWT^*^ RD to CGT: WD^*^, distance walked per RD session^*^
Swank et al. (2020b)	Retrospective investigation of RD with control group	Investigate Ekso RD RGT utilization and its effect on functional outcomes in stroke	Acute stroke: *n* = 96 38L/51R/7Bi Age avg: 62 SCI: *n* = 59 Age avg: 48.2	Ekso RD, ≥5 RD sessions only included, compared to ≥1 CGT session	Timeframe: admission, discharge Conditions: CGT, RD + CGT Clinical: Stroke Rehabilitation Assessment of Movement (STREAM), FIM motor, FIM total	CGT to RD: STREAM at admission and discharge^*^
Nilsson et al. (2014)	Interventional study comparing pre-post effects with no control group.	Investigate the safety, feasibility and functional changes after HAL RD gait training in stroke	Subacute stroke: *n* = 8 4R/4L Age: 39–64	HAL RD, 6–17 sessions, 1 h/session, 5 days per week	Timeframe: BL-Post Conditions: voluntary and autonomous mode Functional: GS, FIM, 10MWT, BBS, FAC, TUG, FMA-LE, NIH stroke Scale, Clinical Outcome Variable Scale-Swedish version, Falls-efficacy Scale Swedish version, BI, EQ-5D, EQ-SD visual analog scales	FAC↑ and 10MWT↑ No significance was calculated
Taki et al. (2020)	Retrospective study comparing pre-post effects with control group	Examine HAL RD clinical outcomes in stroke patients using propensity score matching	Acute stroke: *n* = 108 Age: CGT-73.8, RD 71.4	RD, CGT 3 h/day, for 7 day/week, RD training 3 times/week for 40 min for RD group	Timeframe: BL to post Condition: RD and CGT Clinical: FIM, Brunstrom recovery stage, Modified Rankin Scale	BL to post comparison between RD and CGT: FIM RD^*^
Li et al. (2021)	Randomized clinical trial	Evaluate BEAR-HI RD training on functional, clinical, and biomechanical outcomes in subacute stroke patients	Subacute stroke: *n* = 37 25L/12R Age: 20–65	BEAR-HI RD or CGT, 30 min, 5 times/week for 4 weeks	Timeframe: BL-post Conditions: RD, CGT Functional: 6MWT Clinical: FAC, FMA-LE, MAS Biomechanical: GS, CAD, SL, SLL, gait cycle duration, SWT	RD to CGT: 6MWT,^*^ FMA-LE^*^, gait speed^*^, CAD^*^, SL^*^, and cycle duration^*^ in RD group
Goffredo et al. (2019)	Interventional study comparing pre-post effects with no control group.	Investigate Ekso RD training on functional and clinical outcomes in subacute stroke	Subacute stroke: *n* = 46 24L/22R Age: 18–80	Ekso RD, 12–20 total sessions per patient, 1 h/session	Timeframe: BL-Post Conditions: ambulant and non-ambulant Functional: BI, TCT, FAC, WHS, 6MWT, 10MWT Clinical: WHS, MAS, MI-AD, MI-KE, MI-HI, MI-Lower Limb, MI-Total	BL to post *n* = 32 ambulant: BI^*^, MI-AD^*^, MI-KE^*^, MI-HF^*^, MI-Lower Limb^*^, MI-Total^*^, TCT^*^, FAC^*^, 6MWT^*^, 10MWT^*^, WHS^*^, BL-post *n* = 14 non-ambulant: *n* = 8 regained ambulation: BI^*^, MI-AD^*^, MI-KE^*^, MI-HF^*^, MI-Lower Limb^*^, MI-Total^*^, TCT, FAC^*^, 6WT^*^, 10mWT^*^, WHS^*^ Subset *n* = 6 not ambulatory at post: BI^*^
Molteni et al. (2017)	Interventional study comparing pre-post effects with no control group	Examine Ekso RD effect on functional and clinical effects in stroke	Subacute: *n* = 12 5L/7R Age avg: 43.8 Chronic: *n* = 11 7L/4R Age avg: 55.5	Ekso RD, 12 sessions, 1 h/session, 3 times/week	Timeframe: BL-Post Conditions: chronic patients: only RD training Subacute: RD plus CGT Functional: BI, TCT, FAC, TUG, WHS, 6MWT, 10MWT (sec), 10MWT (steps), 10MWT (m/s) Clinical: MAS-H, MAS-A, MI	BL to 6 sessions subacute: MI^*^, FAC^*^, 6MWT^*^, 10mWT (m/s)^*^ 6 sessions to 12 sessions subacute: MI^*^, TCT^*^, 6MWT^*^ BL to 12 sessions subacute: MI^*^, TCT^*^, FAC^*^, 6MWT^*^, 10MWT^*^, WHS^*^ BL to 6 sessions chronic: MI^*^ 6 sessions to 12 sessions chronic: MI^*^, 10MWT (m/s)^*^, 6MWT^*^ BL to 12 sessions chronic: MI^*^, FAC^*^, 10mWT (m/s)^*^, 6MWT^*^
Molteni et al. (2021)	Randomized clinical trial	Evaluate Ekso RD effect on functional and clinical outcomes for stroke	Subacute stroke: *n* = 75 RD: *n* = 38 CGT: *n* = 37 45L/30R Age: 18–80	Ekso RD, CGT 15 sessions (5 sessions/week for 1 h each)	Timeframe: BL and post Conditions: RD, CGT Functional: 6MWT, TCT, FAC, 10MWT Clinical: MAS-AL, MI-Affected Limb, mBI, WHS	BL to post, RD and CGT: All outcomes showed significant improvements
Mizukami et al. (2016)	Interventional study comparing pre-post effects with no control group	Examine effect of HAL RD in improving functional and clinical outcomes in stroke	Subacute stroke: *n* = 8 5L/3R Age: 26–76	HAL RD, 25 sessions, 20-minute HAL treatment + 40-minute regular PT training/session	Timeframe: BL-Post Functional: speed from 10MWT, GS, 2MWT, FAC, BBS Clinical: FMA	BL to post: MWS^*^, GS^*^, and 2MWT^*^
Watanabe et al. (2017)	Randomized control trial	Examine the effect of HAL RD on different outcomes between conventional and RD training in stroke patients	Subacute stroke: *n* = 24 RD: *n* = 12 5L/7R Age avg: 66.9 *n* = 12 CGT Age avg: 76.8	HAL RD or CGT, 12 sessions, 20 min/session	Timeframe: BL-post, 8–12 weeks of RD/CGT Conditions: CGT, RD Functional: FAC, TUG, 6MWT, MWS Clinical: FMA Biomechanical: CAD, SLL	BL to post CGT: FAC^*^ BL to post 8 weeks CGT: FAC^*^ BL to post 12 weeks CGT: FAC^*^
Yeung et al. (2021)	Randomized clinical trial	Evaluate ankle robot control modes in improving functional outcomes in stroke	Subacute stroke: *n* = 47 23L/24R Age avg: 65.5	Ankle RD, power-assisted ankle robot, (PAAR) and swing-controlled ankle robot (SCAR) 30 min/session, 20 sessions followed by 2 h CGT, CGT only	Timeframe: BL-post Condition: RD + CGT, CGT Functional: FAC, BBS, 10MWT	BL to post within both groups: CAD,^***^, speed^***^, FAC^***^, BBS^***^, 10MWT^***^ PAAR to SCAR: more stairs and faster walking in PAAR^**^
Schröder et al. (2019)	Interventional study comparing pre-post effects with control group	Examine if Ekso GT improves functional and biomechanical outcomes in stroke	Chronic stroke: *n* = 7 5L/2R Age avg: 53	Ekso GT RD and CGT, both groups: 1 h, 16 sessions	Timeframe: BL-Post Condition: RD, CGT Functional: 10MWT, 6MWT Biomechanical: walking symmetry ratio	BL to post RD: 2/3 10mWT^*^, 3/3 6minWT^*^ BL to post CGT: 2/4 10mWT^*^, 1/4 6minWT^*^
De Luca et al. (2020)	Randomized control trial	Evaluate if Ekso GT RD improves psychological wellbeing of patients, QOL, and GI function in stroke	Chronic stroke: *n* = 30 Age avg: 55.1	Ekso RD, and CGT both performed 24 sessions of gait training separately, 1 h/session	Timeframe: BL-Post Condition: RD, CGT Functional: 10MWT, TUG Clinical: Hamilton Rating Scale for Depression, short form Quality of life, FIM, RMI, Constipation Scoring System (CONST) PGWI: Anxiety, depression, General Health, Vitality, Positive wellbeing, self-control Coping Orientation to Problems Experienced (COPE): Social Support, Avoidance, Positive Attitude, Problem Orientation, Tran-scendental	BL to post RD: 10MWT^***^, TUG^***^, CONST^***^, Hamilton Rating Scale for Depression^**^, PGWI^**^, Anxiety^**^, depression^*^, Vitality^**^, General Health^**^, Positive wellbeing^***^, COPE-Social Support^***^, Avoidance^***^, Positive Attitude^***^, Problem Orientation^***^, short form Quality of life^***^, FIM^***^, RMI^***^, CONST^***^ BL to post CGT: COPE Problem Orientation^***^, FIM^***^, CONST^**^, TUG^***^, RMI^***^
Goffredo et al. (2019)	Interventional study comparing pre-post effects with control group.	Evaluate improvements of clinical and functional outcomes using Ekso RD compared to end-effector training and CGT in stroke	Subacute stroke: *n* = 26 11L/15R Age: 18–80	Ekso RD, end effector and CGT 15 ± 2 sessions, 1 h/session	Timeframe: BL-Post Conditions: End-effector training, RD, CGT Functional: TUG, 10MWT, 6MWT, WHS Clinical: MI-affected limb, MAS-AIL mBI, TCT, FAC Biomechanical: Spatial-temporal characteristics	BL to post end-effector training: mBI, MI-affected Limb^*^, TCT^*^, FAC^*^, WHS^*^, TUG^*^, 6MWT^*^ BL to post RD overground: mBI^*^, MI-affected Limb^*^, FAC^*^, WHS^*^, and 10MWT^*^ BL to post CGT: mBI^*^, MI-affected limb^*^, TCT^*^, FAC^*^, WHS^*^, TUG^*^
Yoshimoto et al. (2015)	Interventional study comparing pre-post effects with control group	Examine HAL RD and CGT improvement in functional outcomes in chronic stroke	Chronic stroke: *n* = 18 9L/9R Age avg: 65.1	HAL RD: 8 sessions, 1 h/session, CGT: training once every 1 or 2 weeks, 1 h/session	Timeframe for RD: BL → 4 sessions → post Conditions: RD, CGT Functional: GS, CAD, and # of steps from 10MWT, TUG, FRT, BBS	BL to 4 sessions in RD groups: GS^**^, CAD^**^, TUG^*^, BBS^*^ BL to 8 sessions in RD: GS^***^, CAD^**^, TUG^**^, FRT^**^, BBS^**^
Tanaka et al. (2019)	Interventional study comparing pre-post effects with no control group	Examine biomechanical gait outcomes in chronic stroke using HAL RD	Chronic stroke: *n* = 9 7L/9R Age: 50–85	HAL RD, 6–15 sessions, 1 h/session	Timeframe: BL-post, and 3 months post Functional: 2MWT, 10MWT, FAC, FIM, Brunstrom recovery stage, GS Biomechanical: SLL, CAD	BL to post: GS^*^, SL^*^, CAD^*^ and 2MWT^*^ BL to 3 months post: GS^*^, SLL^*^, CAD^*^ and 2MWT^*^
Yoshimoto et al. (2016)	Intervention case study with pre-post evaluation	Examine functional outcomes in chronic stroke using HAL RD	Chronic stroke: *n* = 1 1L Age: 60–65	HAL RD, 8 sessions, 1 h/session	Timeframe: BL, post, 2 months post Functional: 10MWT, TUG, FRT, 2 Step Test, BBS	BL to post: all outcome ↑ BL to 2 months post: 10MWT and GS ↓ No significance calculated.
Kawamoto et al. (2013)	Intervention study with pre-post evaluation with no control group	Invesigate if HAL RD improves functional and biomechanical outcomes in chronic stroke	Chronic stroke: *n* = 16 7L/9R Age avg: 61	HAL RD, 16 sessions, 20–30 min/session	Timeframe: BL-Post Functional: CAD, # of steps, speed from 10MWT, BBS, TUG	BL to post: GS^*^, BBS^*^, CAD^*^, # of steps^*^
Jyräkoski et al. (2021)	Intervention study with pre-post evaluation with no control group	Evaluate Indego RD effect on functional outcomes in brain injury	Subacute and chronic stroke: *n* = 4 TBI: *n* = 1 4L/1R Age: 30–69	Indego RD, 16 sessions, 1 h per session	Timeframe: BL-post Functional: 6MWT, 10MWT	BL to post: $\frac{3}{4}$ 10 minWT ↑, 4/4 6MWT ↑ No significance calculated
Bortole et al. (2015)	Intervention case series with pre-post evaluation with no control group	Examine the feasibility and safety and clinical outcomes of the H2 RD in stroke	Chronic stroke: *n* = 3 3L Age: 43, 45, 58	H2 RD, 12 sessions, 40 min/session	Timeframe: BL-Post Functional: BBS, TUG, 6MWT Clinical: FMA, Functional Gait Index, BI	BL to post: Subject 1 BBS ↑, Subjects 1 and 3 Functional Gait Index ↑, Subjects 2 and 3 6MWT, TUG, and FMA ↑, Subject 2 BI ↑ No significance calculated
Yeung et al. (2018)	Randomized control trial	Investigate RD AFO on improving clinical and functional outcomes in stroke	Chronic stroke: *n* = 19 10L/9R Age: 45–70	RD AFO, and sham 20-1 h sessions, walking tasks: overground, ascending/descending stairs	Timeframe: BL-Post Conditions: RD AFO, sham Functional: 10MWT, 6MWT, BBS, FAC, FMA, MAS	BL to post: FAC^*^, 10MWT^*^, FMA^*^
Panizzolo et al. (2021)	Intervention study with pre-post evaluation with no control group	Evaluate if Exoband passive RD improves walking distance in ABI	Neurological: *n* = 10 stroke: *n* = 4 Age avg: 68.9 ± 9.2	Exoband passive exoskeleton, 10 sessions, 10 min/session	Timeframe: BL-post Functional: WD, 6MWT Clinical: Borg rate of perceived exertion	BL to post: WD^*^
Kovalenko et al. (2021)	Randomized control trial	Evaluate ExoAtlet RD capability of improving clinical and functional outcomes in stroke	Chronic stroke: *n* = 42 Age: 47–75	ExoAtlet RD, 10 sessions, 1 h/session, botulinum neurotoxin (BNT) injection given after 10 sessions	Timeframe: BL, post-RD (day 12), post BNT (day 33) Conditions: RD, CGT Functional: 10MWT, BBS, RMI Clinical: MAS, Rankin Scale, Visual Analog Scale, TS	BL to mid RD: 10MWT^*^, BBS^*^, TS^*^ Mid to post RD: 10MWT^*^, BBS^*^, TS^*^ BL to post RD to CGT: 10MWT^**^, BBS^**^, TS^**^
**Stroke- Rigid exoskeletons: Biomechanical and physiological outcomes**						
Høyer et al. (2020)	Exploratory study with pre-post evaluation with no control group	Examine if Ekso RD improves biomechanical, functional and clinical outcomes in stroke	Subacute stroke: *n* = 26 Age avg: 54.4 18	Ekso GT RD, 1 h/session, 2–3 times a week for 3 week,	Timeframe: BL-Post (clinical), 3rd session-post (functional) Functional: WT Biomechanical: up-time, number of steps, Borg scale Clinical: MAS	BL to post clinical: MAS^**^ Third session to post functional: WT^***^, up-time^***^, and number of steps^***^
Rojek et al. (2020)	Randomized control trial	Investigate if Ekso GT RD improves biomechanical and functional outcomes in stroke	Chronic stroke: *n* = 44 24L/20R, Age: 55–85	Ekso GT RD, CGT 5 times/week, 45 min/session plus 1 h PT	Timeframe: BL-Post Conditions: RD, CGT Biomechanical: balance, load distribution, COP PL and COP avg Velocity (eyes open and closed) Clinical: RMI, BI	BL to post RD: COP PL and VEL ↑ eyes closed BL to post CGT: COP PL ↑ eyes open BL to post RD and CGT: COP Velocity ↑ eyes open, RMI^*^ and BI^*^
Murray et al. (2015)	Intervention case series with pre-post evaluation with no control	Evaluate Vanderbilt RD controller in biomechanical outcomes for stoke	Chronic hemiplegic *n* = 3 1L/2R Age: 39, 42, 69	Vanderbilt RD, 3 sessions, 30 min/session	Timeframe: BL-Post in each session Functional: 10MWT Biomechanical: GS, SLA, and SLL	BL to post: Improvement in each session, no significance noted or calculated
Murray et al. (2014)	Intervention case study with pre-post evaluation	Evaluate if novel controller in Vanderbilt RD improves biomechanical outcomes in stroke	Subacute stroke: *n* = 1 right side weakness age: 39	Vanderbilt RD, 3 sessions, 10-meter walk, 20–30 min/session	Timeframe: BL-post Functional: GS from 10MWT Biomechanical: SLA, SLL	BL to post: GS, SLA and SLL ↑ No significance calculated.
Buesing et al. (2015)	Randomized control trial	Examine the impact of Stride Management Assist RD on biomechanical gait outcomes in stroke patients	Chronic stroke: *n* = 50 25L/25R Age: 18–85 years	SMAS RD, CGT 18 sessions, 45 min/session	Timeframe: BL, mid, post, 3 months Conditions: RD, CGT Biomechanical: GS, CAD, ST, SL, SLL, SWT, STT, and DST, spatial asymmetry	BL-Mid RD: GS^**^, CAD^**^ BL-Mid RD (Impaired): SL^**^, SLL^**^, STT^**^, DST^**^ BL-Mid RD (non-impaired): ST^**^, SL^**^, SLL^**^, STT^*^, DST^**^ BL-Post RD: GS^**^, CAD^**^, temporal sym^**^ BL-Post RD (impaired): ST^**^, SL^**^, SLL^**^, SWT^**^. STT^**^, DST^**^ BL-Post RD (non-impaired): ST^**^, SL^**^, SLL^**^, STT^**^, DST^**^ BL-Follow up RD: GS^**^ BL-Follow up RD (impaired): SLL^**^, SL^**^, STT^**^, DST^**^ BL-Follow up RD (non-impaired): SLL^**^, STT^**^, DST^**^ Mid-Post RD: GS^*^ Mid-Post RD (impaired): SL^**^, SLL^**^, DST^**^ Mid-Post RD (non-impaired): SLL^**^, STT^**^, DST^**^ BL-Mid CGT: GS^**^ BL-Mid CGT (impaired): SL^**^, SLL^**^ BL-Mid (non-impaired): SL^**^, SLL^**^ BL-Post CGT: GS^**^, CAD^**^ BL-Post CGT (impaired): ST^**^, SL^**^, SLL^**^, STT^**^, DST^**^ BL-Post CGT (non-impaired): ST^**^, SL^**^, SLL^**^, STT^**^, DST^**^ BL-Follow up CGT: GS^**^ BL-Follow up (impaired): SLL^**^ BL-Follow up (non-impaired): SL^**^, SLL^**^ Mid-Follow up (non-impaired): SL^**^
Tan et al. (2018)	Interventional study with no control group	Determine effect of HAL RD training in stroke	Acute stroke: *n* = 8 4L/4R Age: 43–80	HAL RD, 9 sessions, 1 h/session	Timeframe: BL-post Physiological: EMG of VM, HAM, TA, GA, AD, Gmax Clinical: L-FIM, m-FIM, FMA-LE Biomechanical: GS, SL, CAD, AA, HA, KA range of motion	BL to post: lateral synergies^*^, FIM-Locomotion^*^, FIM-Motor^*^, FMA^*^, GS^*^, CAD^*^
Tan et al. (2020)	Interventional study with control group	Evaluate the effects of HAL RD compared to CGT muscle synergy symmetry and clinical outcomes in stroke	Subacute stroke: *n* = 20 10L/10R Age: 40–80	HAL RD, 9 sessions, 20 min/session	Timeframe: BL, 4th session, 7th session, post, Conditions: RD, CGT group Physiological: VM, HAM, TA, GA, AL, Gmax Clinical: L-FIM, m-FIM, FMA	BL to post RD: muscle timing symmetry^*^, FIM-L^*^ FIM-M^*^, and FMA^*^ BL to post CGT: FIM-L^*^ FIM-M^*^, and FMA^*^
Zhang et al. (2020)	Randomized control study	Evaluate RoboCT RD clinical outcomes in stroke	Acute and Subacute Hemiplegic stroke: *n* = 24 Age avg: 51	RoboCT RD, 20 sessions, 30 min CGT, 20 sessions, 30 min	Timeframe: BL-post Conditions: RD, CGT Biomechanical: Manual Muscle Strength Test (MMT) of TA	BL to post RD: MMT^*^ BL to post CGT: MMT^*^ RD to CGT: MMT^*^ for RD
Infarinato et al. (2021)	Interventional study with no control group	Evaluate o-RAGT RD training muscles activation patterns, functional, and clinical outcomes in subacute stroke patients	Subacute stroke: *n* = 8 2L/6R Age: 18–80	Ekso RD, 15 sessions of overground RD training, 1 h/session, 5 times a week	Timeframe: BL to post Functional: 10MWT Clinical: TCT, MAS. MI, FAC Physiological: BS, Co-Contraction, and root mean square from sEMG of TA, GM, RF, and BF	BL to post: MI^*^, FAC^*^, BS^*^ of TA
Kotov et al. (2021)	Randomized study	Examine if ExoAtlet RD is capable of improving functional and clinical outcomes in stroke compared to pedal trainer	Subacute and chronic stroke: *n* = 47 ExoAtlet RD: *n* = 23 MOTO pedal trainer: *n* = 24 18L/29R Age: 18–80	ExoAtlet RD, 5 days/week for 2 weeks, 10–30 min/session using RD in group 1 and using Pedal Trainer in group 2	Timeframe: BL-post Functional: 10MWT, BBS Conditions: RD, MOTO Clinical: MRC, MAS, Modified Rankin Scale, BI, Hauser Ambulation Index Physiological: EMG of TA, MG, Gmax Biomechanical: SLL, cycle duration, GS, CAD, statokinesiogram	BL to post RD: SLL^*^, cycle duration^*^, GS^*^, CAD^*^, curve in statokinesogram eyes closed^*^, BI^*^ BL to post MOTO: statokinesograph length eyes closed^*^ RD to MOTO: MRC^*^, BBS^*^, Hauser Ambulation Index^*^, 10MWT^*^, BI^*^, length and area of statokinesiogram eyes open^*^
Zhu et al. (2021)	Interventional study with no control group	Evaluate the effect of Ekso RD on neuromuscular co-ordination in stroke	Chronic stroke: *n* = 12, 5 participated in longitudinal RD study (2F/10M), HC: *n* = 11 (5F/6M) Age: at least 18 years	Ekso 1.1^TM^ RD, 10–15 sessions, 50 min /session. The therapist controlled the modes throughout therapy	Timeframe: BL-post Conditions: With and Without RD, HC Functional: 10MWT, 6MWT, TUG Physiological: energy expenditure, EMG of TA, MG, VM, BF-Long head, SEMI-T, Gmax, GM, muscle synergy and motor modules Biomechanical: AA, KA, HA	stroke vs. HC: Muscle synergy pattern: 4 modules HC and non-paretic side stroke, 3 modules stroke paretic leg, BL to post RD: 10MWT^*^, 6MWT^*^, ↑ synergy pattern after training
Lee et al. (2019)	Randomized control trail	Evaluate the effects of GEMS RD biomechanical, physiological, clinical, and functional outcomes in stroke	Chronic stroke: *n* = 26 15L/11R Age avg: 62	GEMS RD, 10 sessions treadmill or overground RD training, CGT no RD, 45 min/session	Timeframe: BL-post Conditions: RD, CGT Biomechanical: GS, CAD, SLL and BS Physiological: bilateral sEMG of RF, BF, TA, GA, MG and cardiopulmonary metabolic efficiency (CPME) Clinical: FMA, FES Functional: BBS	BL to post RD: GS^*^, CAD^*^, SLL^*^, gait sym ratio^*^, RF^*^, BF^*^, TA^*^, GA^*^, CPME^*^ BL to post CGT: GS^*^, CAD^*^, SLL^*^, RF^*^ RD to CGT: SLL^*^, gait sym ratio^*^, EMG of RF^*^, GS^**^, CAD^**^, BF^***^, TA^***^, GA^***^, CPME^*^ for RD
Li et al. (2015)	Interventional study with no control group	Examine clinical, biomechanical and physiological outcomes using RLO leg in stroke patients	Chronic stroke: *n* = 3 1L/2R Age: 53, 61, 62	RLO RD, 15 sessions, 1 h/session	Timeframe: BL-post Clinical: BBS, LE-FMA Physiological: EMG of RF, TA, BF, GM Biomechanical: CAD, SL, GS	BL to post: BBS, LE-FMA, CAD, SL, and GS ↑, ankle symmetry, MG and BF ↑ No significance calculated
**Stroke- Rigid exoskeletons: Neurological outcomes**						
Calabrò et al. (2018)	Randomized clinical trial	Examine the effect of Ekso RD gait training on cortical, functional, and physiological outcomes in stroke	Chronic stroke: *n* = 40 22L/18R Age avg: 67	Ekso RD, 40 sessions, 1 h/session	Timeframe: BL-post Conditions: RD +CGT, CGT Cortical: CSE and SMI Functional: 10MWT, TUG Clinical: RMI Physiological: sEMG of TA, SO, RF, and BF Biomechanical: stance/swing ratio, gait quality index, CAD, gait cycle duration	RD to CGT: activity of RF^*^, BF^*^, SO^*^ RMI^*^, TUG^*^, stance/swing ratio^**^, CSE^**^, SMI^**^, FPEC^**^, gait quality index^***^, CAD^***^, gait cycle duration^***^, 10MWT^***^in RD
Molteni et al. (2020)	Randomized crossover trial	Examine the effects of short term Ekso GT RD training on neuroplastic modulation in chronic stroke	Chronic stroke *n* = 9 4R/5L Age: 30–75	Ekgo GT RD training and overground CGT, 1 h/session	Timeframe: Pre-post training Condition: RD, CGT Cortical: Coherence for alpha1, alpha2, and beta frequencies. Node strength and betweenness centrality	RD to CGT: Both groups node strength ↑ in alpha1, alpha2, and beta bands, betweenness centrality ↓ in alpha2 over vertex in left hemisphere stroke In Right hemisphere stroke, node strength ↑ in alpha, alpha2 over the contralesional sensorimotor area and ipsilesional prefrontal area in RD at Post
Jayaraman et al. (2019)	Randomized clinical trial	Evaluate Honda Stride management assistant RD gait outcomes in stroke compared to conventional training	Chronic stroke: *n* = 50 25L/25R Age: 18–85	Honda RD, 18 sessions, 45 min/session	Timeframe: BL, mid, post, and 3 months post Condition: RD, CGT Functional: 10MWT, 6MWT, BBS, Sit to Stand Test Clinical: LE-FM, Cortical: CME of paretic RF, TA, lateral hamstrings	BL-mid, post, and 3 months post RD: 10MWT^*^, 6MWT^*^, BBS^*^, FMA-LE^*^, CME of RF^*^ (only at post) BL-mid, post, and 3 months post CGT: 10MWT^*^, 6MWT^*^, BBS^*^, FMA-LE^*^, CME of lateral hamstrings^*^ (only at post), CME of TA^*^ (only at post) RD to CGT: 6MWT^*^, BBS^*^
**Stroke- Soft exoskeletons: Functional and clinical outcomes**						
Haufe et al. (2020)	Interventional study with no control group	Examine the effects of Myosuit RD functional outcomes for stroke	Chronic stroke *n* = 2 1L/1R SCI: *n* = 4 Other: *n* = 2 Age: 18–80	Myosuit RD, 5 total sessions, 45 min/session	Timeframe: BL-post Functional: 10MWT GS, 2 minWT WD, Daily step count, Borg scale	BL to post: GS for 5/8 participants^**^
Monticone et al. (2013)	Randomized controlled trial	Evaluate Regent RD on improving functional and clinical outcomes between RD and CGT in stroke	Subacute stroke: *n* = 60 Age: 40–75	Regent RD, CGT, 20 sessions, 30 min	Timeframe: BL-post Conditions: RD, CGT Functional: 6MWT, BBS, BI Clinical: FIM	BL to Post: 6MWT, BBS RD to CGT: 6MWT^***^
**Stroke- Soft exoskeletons: Neurological outcomes**						
Saenko et al. (2016)	Intervention study with pre-post evaluation with no control group	Examine the effects of regent RD cognitive outcomes in stroke patients	Subacute and chronic stroke: *n* = 14 7L/7R Age avg: 50.3	Regent RD, 10 sessions	Timeframe: BL-post Cortical metric: fMRI Clinical metric: FMA Functional metric: 10MWT	BL to post: 10MWT^*^, activation zones of the IPL^***^↓, activation zones of the Primary sensorimotor^***^↑ and SMA^***^ ↑
Poydasheva et al. (2016)	Intervention study with pre-post evaluation with no control group	Evaluate the capability of nTMS to assess changes in gait cortical control using SEC in poststroke patients	Chronic stroke: *n* = 14 7L/7R Age avg: 53	Regent RD, 10 sessions	Timeframe: BL-post Cortical: nTMS Functional: 10MWT Clinical: FMA	BL to post: 10MWT^*^, nTMS latency of response in ankle symmetry^*^

^*^Level of *p* < 0.05 significance, ^**^level of *p* < 0.01 significance, ^***^Level of *p* < 0.001 significance; ↑, increase; ↓, decrease; BL, baseline.

The review results have been divided based on the diagnosis [cerebral palsy (CP), traumatic brain injury (TBI), and stroke]. The review is further divided based on outcomes metrics (functional and clinical, physiological and biomechanical, and neurological). The soft RD's were reviewed separately from rigid exoskeletons. Table 2 describes the known technical characteristics of all the exoskeletons reviewed in this article. Table 1 describes the studies reviewed in this article. Abbreviations are listed in Table 3.

**Table 2:** List of exoskeletons.

**RD name**	**Known technical characteristics of the RD**		**Diagnosis**
**Rigid exoskeletons**			
HAL-Hybrid Assistive Limb (Cyberdyne Inc.)	Hip and knee joints are bilaterally actuated. The HAL has three control systems comprising the Cybernic Voluntary Control (CVC), Cybernic Impedance Control (CIC), and Cybernic Autonomous Control (CAC). The CVC mode assists patients' motion triggered by their EMG in the hip and knee extensor and flexor muscles. An assistive torque was given to each joint according to the detected EMG, with modulation by magnitude, timing of agonist activity, and balance between agonist and antagonist activities. CAC mode provides assistive torque leg trajectories based on postural cues and sensor shoe measurements. CIC mode provides torque to compensate for frictional resistance of the motor based on joint motion. CIC mode does not provide torque assistance for dictating joint trajectories	Multijoint	Chronic diplegic/quadriplegic CP (Ueno et al., 2019; Kuroda et al., 2020)
		Multijoint	Acute (Nilsson et al., 2014; Taki et al., 2020)/chronic stroke (Kawamoto et al., 2013; Tanaka et al., 2019)
	The single-leg version of the HAL is a wearable robot for patients with hemiplegia that has the cybernic voluntary control mode and the cybernic autonomous control mode.	Multijoint	Acute stroke (Tan et al., 2018; Tanaka et al., 2019)/subacute (Mizukami et al., 2016; Watanabe et al., 2017; Tan et al., 2020)/chronic stroke (Yoshimoto et al., 2015, 2016)
CPWalker	The CPWalker rehabilitation device is composed of an exoskeleton linked to a walker that provides support and balance to the user during over-ground training. There are three training modes: position control mode—the robot guides a prescribed gait pattern to the user's lower limbs; Impedance control modes—in this mode, assistance by the robot is provided as needed by the user to achieve the desired gait pattern; Zero-force control mode—in this mode, the trajectory reference is not given, and the user moves the legs with minimal resistance from the device. It is used with users with enough motor control (acquired with the previous modes) but poor balance, so the device provides stability while the user performs the gait pattern	Multijoint	Chronic, spastic diplegic CP (Bayón et al., 2016, 2018)
Novel exoskeleton for crouch gait (Ultraflex Systems)	Wearable robot provides on-demand assistive torque at the knee joint to facilitate knee extension during walking while preserving (or enhancing) muscle activity of the user. Knee angle, FSR, and joint torque signals are input into a feedback control system to control the knee joint torque	Single joint	Chronic, diplegic CP (Lerner et al., 2017a,b; Bulea et al., 2018)
Adaptive Ankle	The RD includes a motor assembly, and an ankle pulley mounted in-line with the ankle joint. A proportional joint-moment control scheme, developed to account for stride-to-stride variability, provided plantar-flexor assistance proportional to the real-time biological ankle joint moment using force sensors placed under the forefoot	Single joint	Diplegic CP (Fang et al., 2020)
Ekso™ (version 1.1) and Ekso GT™ (version 1.2) (Ekso Bionics)	Hip and knee joints are bilaterally actuated. The software control included ProStep Plus™—each step was triggered by the subject's transfer load from one leg to the other and assistance is provided as needed; Bilateral Max Assist—the amount of power contribution to the legs during walking was totally provided by the robot	Multijoint	Acute (Nolan et al., 2018)/chronic TBI (Karunakaran et al., 2020a,b)
	Hip and knee were bilaterally actuated. The software control included ProStep Plus™ (each step was triggered by the subject's transfer load from one leg to the other) and Bilateral Max Assist (the amount of power contribution to the legs during walking was totally provided by the robot)	Multijoint	Acute (Pournajaf et al., 2019; Lefeber et al., 2020; Nolan et al., 2020; Swank et al., 2020b; Karunakaran et al., 2021)/subacute (Goffredo et al., 2019; Høyer et al., 2020; Infarinato et al., 2021; Molteni et al., 2021)/chronic stroke (Molteni et al., 2017; Calabrò et al., 2018; Schröder et al., 2019; De Luca et al., 2020; Rojek et al., 2020; Zhu et al., 2021)
BEAR-HI (Shenzhen MileBot Robotics Co., Ltd.)	Hip, knee, and ankle are actuated in the sagittal plane. The RD has a training mode and an intelligent mode. For the training mode, stride frequency could be changed within 3% of the set gait cycle frequency. In the intelligent mode, stride frequency could be adjusted in real-time to achieve synchronization of human-robot interaction. The assistance was provided based on the assist-as-need concept	Multijoint	Subacute stroke (Li et al., 2021)
Wearable ankle	Force sensitive resistors (FSR) were used to identify gait phase and the ankle was actuated to provide support for dorsiflexion/plantarflexion	Single joint	Subacute stroke (Yeung et al., 2021)
Indego (Parker Hannifin Corp)	Actuated hip and knee. The robot has two modes: Therapy+ and Motion+. In Therapy+, hip flexion initiates steps with trajectory determined by the user with adjustable levels of assist during stance/swing. In Motion+, postural changes triggered the steps with predetermined step and full or variable assist	Multi joint	Subacute and chronic stroke, TBI (Jyräkoski et al., 2021)
H2	Hip, knee, and ankle joints are actuated. Foot switches, potentiometers, hall effect sensors, and strain gauges were used to detect different phases of gait. An assistive gait control algorithm was developed to create a force field along a desired trajectory, only applying torque when patients deviate from the prescribed movement pattern	Multi joint	Chronic stroke (Bortole et al., 2015)
Robot-assisted ankle-foot-orthosis	Ankle was actuated. FSR and inertial measurement unit (IMU) were used to detect gait phases to provide dorsiflexion assistance	Single joint	Chronic stroke (Yeung et al., 2018)
Exoband	Exoband is a passive hip assistive device. The device includes three main components: a waist belt and two thigh parts connected to the waist belt by means of two elastic elements, one for each leg. When the hip extends the elastic element stretches, thus storing elastic mechanical energy. When the leg starts to accelerate forward the elastic element initiates to shorten and applies a force in parallel with the hip flexor muscles, ultimately assisting the user's gait. The amount of force applied to the user can be changed by varying the length of the ratchet strap	Single joint	Chronic stroke (Panizzolo et al., 2021)
ExoAtlet (Exoatlet Global S.A.)	ExoAtlet is actuated at the hip and the knee joints. Patients can control the level of support they receive from the exoskeleton through various types of control systems. These include tablets, buttons on the control handles or smart crutches	Multi joint	Subacute (Kotov et al., 2021) and chronic stroke (Kotov et al., 2021; Kovalenko et al., 2021)
Vanderbilt	The exoskeleton incorporates four control actuators that provide sagittal-plane torques bilaterally at hip and knee joints. IMU's are used to detect step initiation and assistance is provided as needed.	Multi joint	Subacute/chronic stroke (Murray et al., 2014)
Stride Management Assist (SMAS) system (Honda R&D Corporation^®^)	This device provides independent assistance with hip flexion and extension for each leg to increase step length. The SMAS control architecture uses a mutual rhythm scheme to influence the user's walking patterns. The SMAS control law uses neural oscillators in conjunction with the user's CPG to synchronize itself with user input. Angle sensors embedded in the SMAS actuators detect the user's hip joint angles throughout the gait cycle. These angles are input to the SMAS controller, which calculates hip joint angle symmetry. The SMAS then generates assist torques at specific instances during the gait cycle to regulate these walking patterns	Single joint	Chronic stroke (Buesing et al., 2015; Jayaraman et al., 2019)
UG0210 (Hangzhou RoboCT Technology Development Co., Ltd)	Hip, and knee are actuated	Multi joint	Acute and subacute stroke (Zhang et al., 2020)
GEMS-Gait Enhancing and Motivating System (Samsung Advanced Institute of Technology)	The GEMS torque assistance units consist of angular sensors and actuators that work on bilateral hip joints. The GEMS can provide assist torque and power around the bilateral hip joints for both extension and flexion during walking	Single joint	Chronic stroke (Lee et al., 2019)
RLO, (Tibion Corporation)	The RLO activated and provided forward propulsion when the participants generated enough force on their paretic knee. The device had an internal sensor that detected the wearer's foot pressure. The RLO provided assistance with extension, controlled flexion, and free movement. Device settings includes changing threshold (the minimum force to activate the device), assistance (the percentage of body weight provided through the limb during extension in the stance phase of the gait cycle), and resistance (level of resistance during flexion on the stance phase of the gait cycle)	Single joint	Chronic stroke (Li et al., 2015)
**Soft exoskeletons**			
ReWalk ReStore™ (ReWalk Robotics, Inc.)	The device consists of motors worn at the waist that generate mechanical forces that are transmitted by cables to attachment points located proximally on a functional textile worn around the calf and distally on a shoe insole to provide dorsiflexion assistance to the ankle	Single Joint	Chronic stroke (Awad et al., 2020)
Myosuit (MyoSwiss AG)	Two adjustable polymer springs cross the hip joints to passively assist hip and actuated knee help with knee movements during gait. Gait events and joint angles were estimated from IMU data. Assistance during gait can be customized to the participant deficits and gait phases as needed	Multi joint	Chronic stroke (Haufe et al., 2020)
Regent	The Regent Suit consists of supporting elements (vest, shorts, knee caps, and foot straps) made of synthetic materials, and a set of elastic loading elements equipped with located fixtures (metal spring hooks) and regulating and locking buckles. There are three sizes of the suit and, for each size, the volume of the vest and shorts can be further adjusted by means of zips sewn on the supporting elements. The elastic elements are fastened to the outer surface of the supporting elements along the patient's body and lower limbs, and not only create a central load on the body and leg muscles, but also allow postural corrections as well as providing for body rotation, stoop, and stretch, which helps to reduce pathological muscular synergisms	Multi joint	Subacute and chronic stroke (Monticone et al., 2013; Poydasheva et al., 2016; Saenko et al., 2016)

**Table 3:** Abbreviations.

Biomechanical and physiological			
AA/HA/KA	Ankle angle/hip angle/knee angle	MMT	Manual muscle strength test
AD/AL	Adductor/abductor longus	PL	Path length
BF	Biceps femoris	RF	Rectus femoris
BS	Bilateral symmetry	SL/SLL	Step length/stride length
CAD	Cadence	SLA	Step length asymmetry
COP	Center of pressure	SEMI-T	Semitendinosus
EMG/sEMG	Electromyogram/surface electromyogram	SO	Soleus
GA/GM	Gastrocnemius/gastrocnemius medialis	SWT/ST/STT	Swing time/step time/stance time
Gmax	Gluteus maximus	SW	Step width
HAM	Hamstring muscle group	TA	Tibialis anterior
IDS/TDS/DST/SST	Initial/terminal/double support time/single support time	TVP	Total vertical pressure
KF/KE	Knee angle/flexion/extension	VL/VM	Vastus lateralis/medialis
MG	Medial gastrocnemius	WT	Walking time
**Clinical**			
COPM	Canadian Occupational Performance Measure	MRC	Medical research council lower limb muscle strength scale
FAC	Functional amblation categories	PCI	Physiological cost index
FMA-LE	Fugl-Meyer assessment-lower extremity	PGWI	Psychological wellbeing index
GMFCS/GMFM	Gross motor function measure	RMI	River mobility index
L-FIM	Locomotor functional independence measure	TS	Tardieu scale
m-FIM/L-FIM	motor/locomotor-functional independence measure	WHS	Walking Handicap Scale
MI-AD/HF/KE	Motricity index-ankle dorsiflexion/hip flexion/knee extension (hemiplegic side)		
**Cortical**			
CSE	Cortico-spinal excitability	nTMS	Navigated transcranial magnetic stimulation
EEG	Electroencephalogram	SMA	Supplementary motor area
fMRI	Functional magnetic resonance imaging	fNIRS	Functional near-infrared spectroscopy
		SMI	Sensorimotor integration
**Functional**			
10MWT	10-m walk test	FGS/GS/MWS/SSWS	Fast/gait speed/max/self-selected
25MWT	25-m walk test	FRT	Functional reach test
25FWT	25-foot walk test	SCT	Stair climb test
2MWT	2-minute walk test	TCT	Trunk control test
30CST	30 s sit to stand test	TUG	Timed up and go
6MWT	6-min walk test	WD	Walking distance
BBS	Berg Balance Scale		

### 3.1. Cerebral palsy

**Figure 4:**
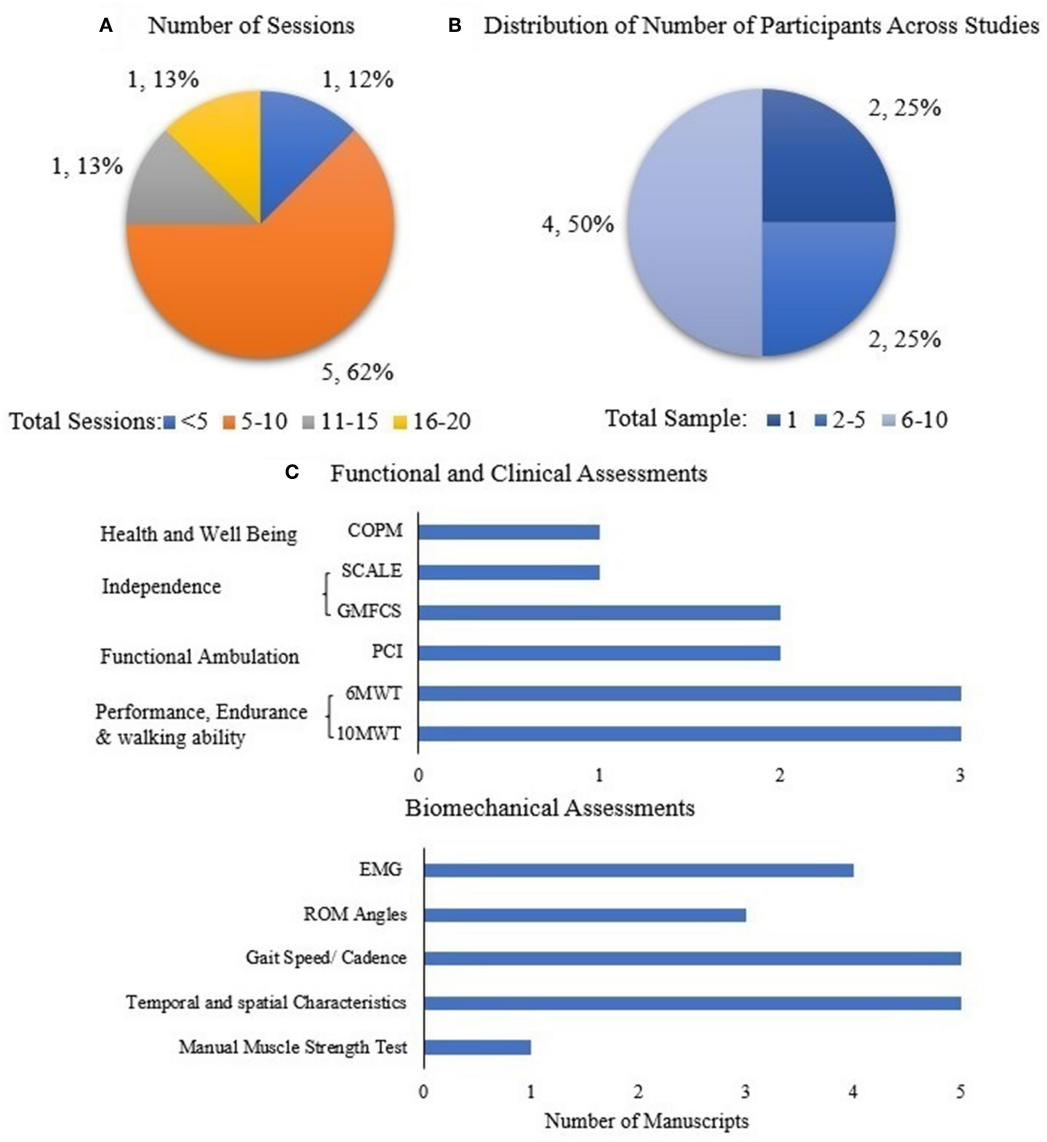
**(A)** Number of sessions across all studies and **(B)** distribution of number of participants across all studies in CP population. Data is represented as the number of studies and as percentage with respect to the total number of studies. **(C)** Assessments used across all studies in CP population.

RD sessions across studies were quantified for the cerebral palsy (CP) population to demonstrate the dosing effect, and is shown in Figure 4A. There is variability across these limited studies in terms of dosing (number of sessions); 62% of the studies were between 5 and 10 sessions. The distribution of number of participants across all studies in CP was quantified in order to understand the generalizability and impact of RD evidence (Figure 4B).

Figure 4C shows the distribution of functional and clinical, and biomechanical assessments across all studies (case study, intervention) in the CP Population. In the limited number of studies in CP population, the most widely used outcomes are performance, endurance and walking ability (10MWT, 6MWT, TUG), as well as spatial-temporal characteristics and cadence.

#### 3.1.1. Rigid exoskeletons

##### 3.1.1.1. Biomechanical and physiological outcomes

Utilization of RD (HAL) gait training in children and adults with CP was evaluated. The preliminary analysis demonstrated improvements in functional and biomechanical outcomes such as gait speed, step length, and cadence in children and adults with no change in GMFM (Ueno et al., 2019). A case study on clinical and physiological metrics also showed that 6 minute walk test (6MWT), gross motor functional measure (GMFM), and Canadian Occupational Performance Measure (COPM) increased, while Physiological Cost Index (PCI) declined after the RD (HAL) intervention (Kuroda et al., 2020).

Similar results were seen while using RD (CPWalker) to restore ambulation. An improvement in biomechanical metrics including step length (Bayón et al., 2016), spatial-temporal parameters (Bayón et al., 2018), and cadence (Bayón et al., 2016), as well as functional and clinical outcomes, such as speed (Bayón et al., 2016, 2018), D and E dimensions (assessed together) of the GMFM-88 scale (Bayón et al., 2018), endurance (6MWT) (Bayón et al., 2018), and strength at the hip and knee (Bayón et al., 2018).

Lerner et al. (2017a) designed a novel RD (ultraflex system) that provides on-demand assistance for knee extension while preserving (or enhancing) muscle activity of the user in CP patients to improve crouch gait. The results from an initial evaluation showed an increase in peak knee extension (Lerner et al., 2017b). The powered exoskeleton significantly altered lower extremity kinematics and reduced the amount of crouch compared to the baseline (BL) condition, resulting in a gait trajectory similar to normal walking (Lerner et al., 2017b). Lerner et al. showed that the knee extension RD for crouch gait increased vastus lateralus (VL) and semitendinosus (SEMI-T) activity during swing and stance respectively on both the affected and unaffected limb (Lerner et al., 2017b). The variability in VL and SEMI-T were low after continued use of this RD (Bulea et al., 2018).

Research on bilateral ankle (Adaptive Ankle) RD showed that participants improved their walking speed and stride length with a corresponding increase in soleus (SO) and VL muscle activity, where SO activity was 39% similar to unimpaired individuals after RD training (Fang et al., 2020).

### 3.2. Traumatic brain injury

**Figure 5:**
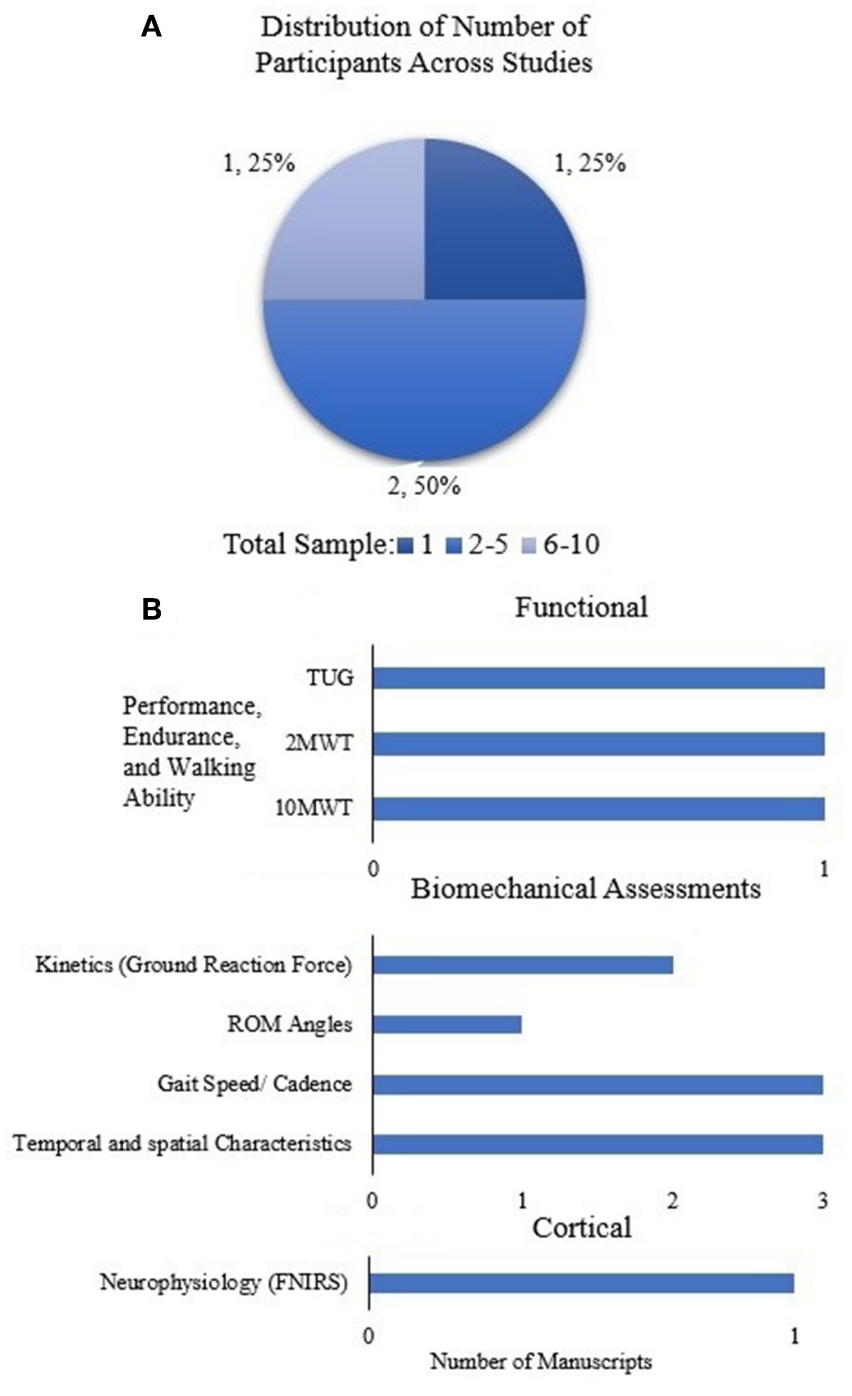
**(A)** Number of participants across all studies in TBI. Data is represented as the number of studies and as percentage with respect to the total number of studies. **(B)** Assessments used across all studies in TBI population.

RD sessions across studies were quantified for the TBI population to demonstrate the dosing effect, and were found to be 12 sessions across the limited number of pre-clinical studies. The distribution of number of participants across all studies in TBI was quantified in order to understand the generalizability and impact of RD evidence (Figure 5A).

Figure 5B shows the distribution of functional and clinical, biomechanical, and cortical assessments across all studies (case study, intervention) in the TBI population.

#### 3.2.1. Rigid exoskeletons

##### 3.2.1.1. Biomechanical outcomes

###### 3.2.1.1.1. Effects of RD in individuals with acute TBI

A case study by Nolan et al. (2018) showed that 4 weeks of RD (Ekso) training in a single young adult with acute TBI had a therapeutic effect after utilizing RD. The participant had a consistent prolonged stance phase bilaterally and performed a more symmetrical gait cycle. RD training resulted in reduced joint angle variability, increased plantar flexion and dorsiflexion, and increased bilateral symmetry, but with decreased walking speed, step length, and swing time (Nolan et al., 2018). There was also an increased compensatory mechanism of hip circumduction.

###### 3.2.1.1.2. Effects of RD in individuals with chronic TBI

TBI research by Karunakaran et al. (2019, 2020a) evaluated the effect of 4 weeks of RD training on gait mechanisms in adolescents and adults with chronic ABI. The results showed that there could be potential long-term effects of improved linearity of loading during initial double support, healthy bilateral loading characteristics, improvement in spatial symmetry, swing time, stance time, and step length with an associated increase in speed, due to RD gait training (Karunakaran et al., 2019, 2020a).

##### 3.2.1.2. Neurological outcomes

###### 3.2.1.2.1. Effects of RD in individuals with chronic TBI

The same group also evaluated the neurophysiological response to RD training in a case study with a participant diagnosed with TBI (Karunakaran et al., 2020b). The results showed that at follow-up there was decreased activity in motor cortex, pre-motor cortex, and supplementary motor area (SMA) with corresponding improvement in gait and balance [improved gait speed and timed up and go (TUG)], suggesting that the participant required less attentional resources to perform the walking task (Karunakaran et al., 2020b).

### 3.3. Stroke

**Figure 6:**
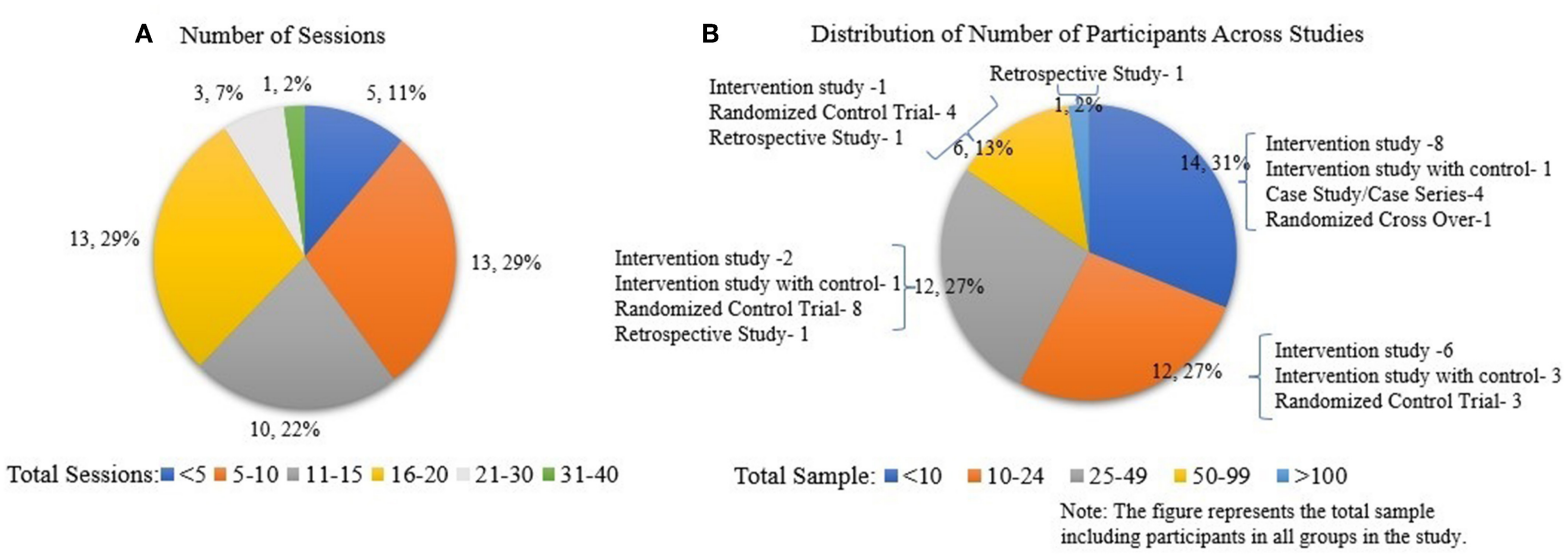
**(A)** Number of sessions across all studies and **(B)** distribution of number of participants across all studies in stroke population. Data is represented as the number of studies and as percentage with respect to the total number of studies.

RD sessions across studies were quantified for the stroke population to demonstrate the dosing effect, and are shown in Figure 6. There is variability across studies in terms of dosing (number of sessions); 80% of the studies were between 5 and 20 sessions. The distribution of the number of participants across all studies in stroke was quantified in order to understand the generalizability and impact of RD evidence. Eighty five percent of the studies had a sample of < 50 participants. This includes intervention and control groups (Figure 6).

**Figure 7:**
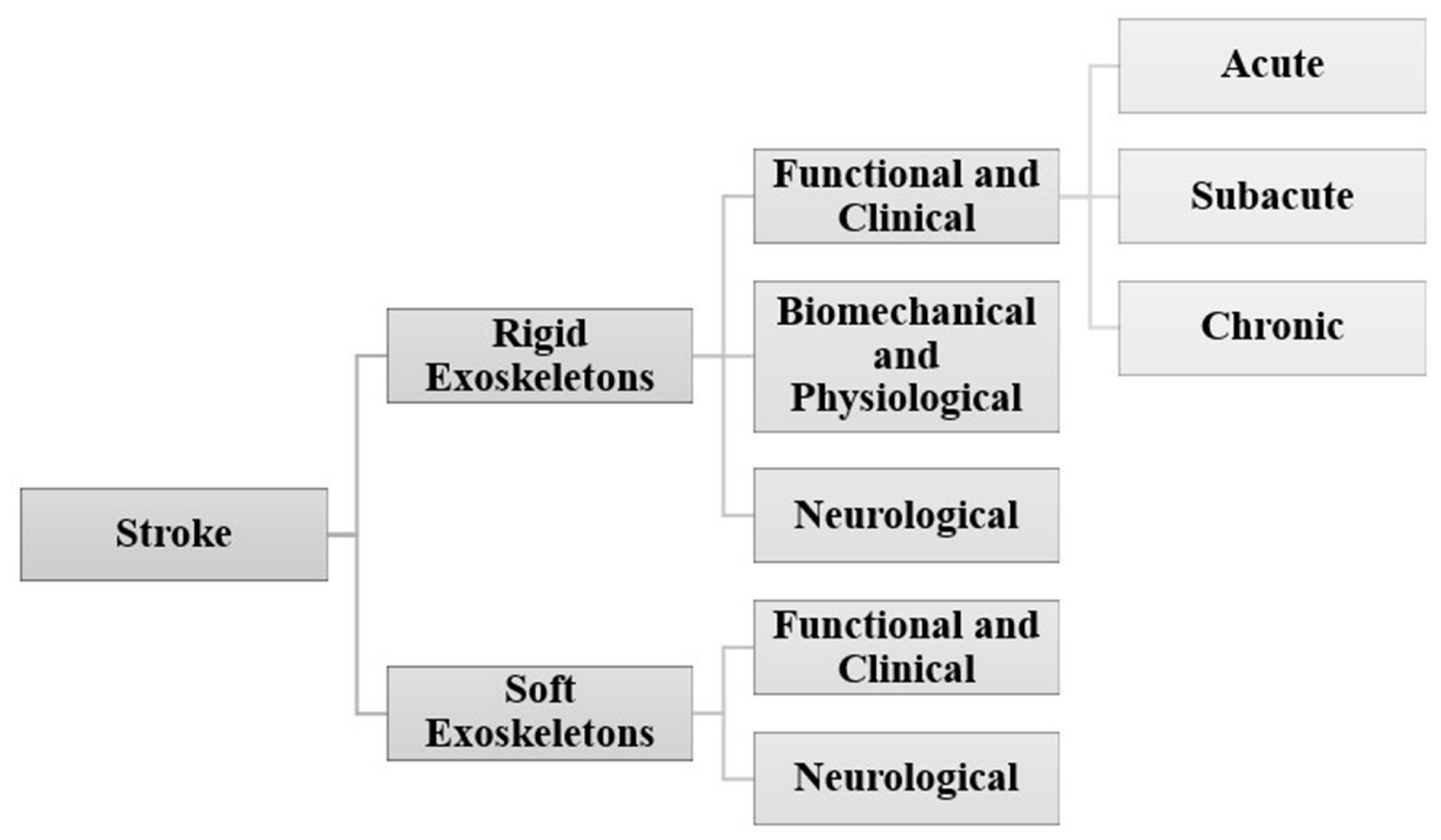
Organization of stroke research.

Figure 7 shows the organization of stroke research in this paper.

#### 3.3.1. Rigid exoskeletons

**Figure 8:**
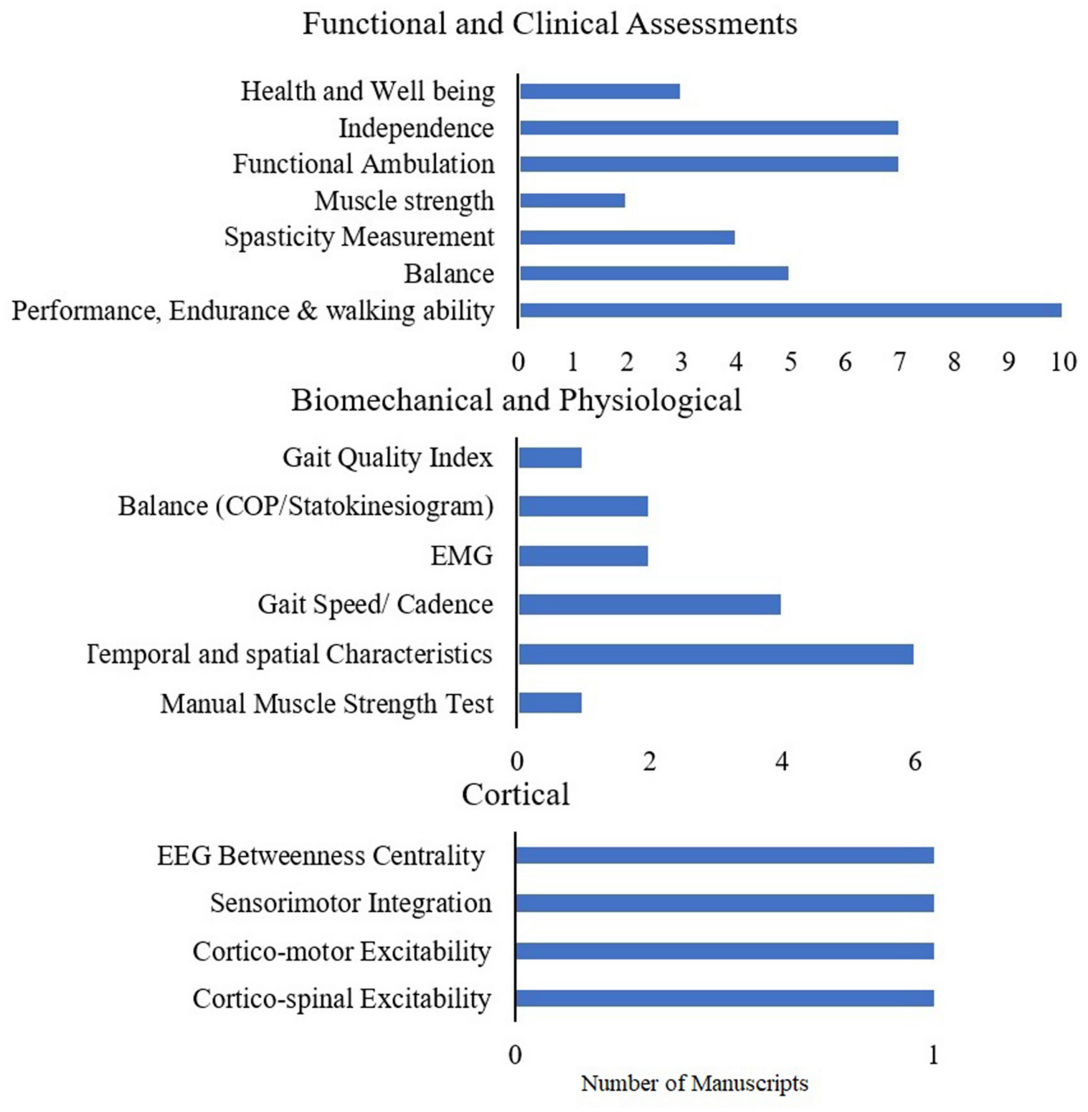
Assessments used in randomized control trails and randomized cross-over trials in stroke.

Figure 8 shows the different outcomes used in the RCTs and the randomized crossover trial for rigid exoskeletons. Most of the RCTs in stroke focus on understanding the effects of RDs, using mostly functional and clinical measures, with very few in other domains. Table 4 shows the distribution of functional and clinical assessments across all studies (RCT, case study, intervention, retrospective, and cross over). The most widely used outcome measures across all studies were performance, endurance and walking ability measures (10MWT, 6MWT, TUG), followed by balance (BBS) and functional ambulation (FAC). Compared to functional outcomes, only a limited number of studies evaluated biomechanical and cortical outcomes. The most widely used biomechanical outcomes are spatial-temporal characteristics and cadence.

**Table 4:** Distribution of studies based on functional and clinical assessment categories across all studies for Rigid RD.

**Category**	**Outcomes**	**Total studies**
Performance, endurance and walking ability	10 m walk test	**25**
	6 min walk test	**13**
	Timed up and go	**10**
	2 min walk test	2
Balance	Berg balance scale	**12**
Spasticity measurement	Ashworth	8
	Tardieu	1
Muscle strength	Mortricity Index	5
	Medical Research Council Lower Limb Strength Scale	1
Functional ambulation	Functional ambulation category	**10**
	Fugl-Meyer assessment	5
	Hauser ambulation index	1
	Clinical outcome variable scale-Swedish version	1
	Functional Gait Index	2
	Borg rate of perceived exertion	2
Independence	Functional independence measure	8
	Barthel Index	8
	Trunk control test	6
	Rankin scale	2
	Functional reach test	2
	Walking handicap scale	2
	Rivermead mobility index	1
	Brunstrom recovery stage	1
Health and wellbeing	EQ visual analog scale	2
	Hamilton Rating Scale for Depression; constipation score; psychological wellbeing score	1
	FES	1
	NIH stroke scale	1

Descriptions regarding the functional and clinical outcomes listed below can be found in reference (Lab SRA, 2023). The bold represent the most used metrics.

##### 3.3.1.1. Functional and clinical outcomes

###### 3.3.1.1.1. Effects of RD in individuals with acute stroke

A retrospective study showed that individuals with acute stroke who received RD (Ekso) training walked twice the distance compared to the standard of care/conventional gait training (SOC/CGT) group during their inpatient physical rehabilitation, though both groups received the same duration of training (time spent in inpatient rehabilitation training session) and similar dosing (Nolan et al., 2020). The RD group also increased their motor FIM score (change from admission to discharge) and motor FIM efficiency compared to the SOC group, though both groups were matched for admission motor FIM scores (Nolan et al., 2020). Similar study evaluating the therapeutic effect on functional ambulation in adults with acute stroke after RD (Ekso) gait training (Karunakaran et al., 2021). The results showed that RD provided high dose training and that there were significant improvements in 10 meter walk test (10MWT), 6MWT, and TUG at follow-up compared to baseline (Karunakaran et al., 2021). Utilization of RD (Ekso) in 96 individuals with acute stroke showed that participants increased their “walk” time and number of steps from session 1 to 5, followed by a plateau from session 5 onwards (with most sessions lasting about 20 mins). Significant differences were observed in Stroke Rehabilitation Assessment of Movement, but weren't observed in FIM in this study (Swank et al., 2020b). In another set of studies, the HAL RD system provided intensive, repetitive gait training in hemiparetic patients in people with acute stroke. All patients improved their walking ability during the training period, as reflected by the 10MWT (from 111.5 to 40 s in median) and the Functional Amblation Categories (FAC) (from 0 to 1.5 score in median) (Nilsson et al., 2014). Similarly, there were significant differences in the Brunstrom recovery stage, FIM total, and FIM motor subscore in the RD (HAL) group compared to SOC at discharge, though there were no significant differences on rehabilitation admission between the groups (Taki et al., 2020). There were also no significant differences in the global disability and score change, defined by modified Rankin Scale, between the groups (Taki et al., 2020). Research on the effects of 4 weeks of BEAR-H1 RD training with conventional training in acute and subacute stroke (Li et al., 2021) showed that there were significant improvements in 6MWT, Fugl-Meyer Assessment-Lower extremity (FMA-LE), gait speed, cadence, step length, and cycle duration at follow-up compared to baseline as well as in the RD group compared to conventional training group with no change in FAC and Modified Ashworth Scale (MAS) between groups (Li et al., 2021).

###### 3.3.1.1.2. Effects of RD in individuals with subacute stroke

Research using RD (EKSO) has shown significant improvements in functional outcomes (6MWT (Molteni et al., 2017; Goffredo et al., 2019), and 10MWT (Molteni et al., 2017; Goffredo et al., 2019), as well as in clinical outcomes [Barthel Index (BI) (Goffredo et al., 2019), Motricity Index (MI) (Molteni et al., 2017; Goffredo et al., 2019), FAC (Molteni et al., 2017; Goffredo et al., 2019), and Walking Handicap Scale (WHS) (Goffredo et al., 2019)], but no change was observed in the MAS (Goffredo et al., 2019) and the Trunk control test (TCT) (Molteni et al., 2017; Goffredo et al., 2019) in individuals with subacute stroke. Also, clinical trials in individuals with sub-acute stroke showed that 6MWT, MI-Affected Limb, 10MWT, mBI, MAS-Affected Limb, FAC, and WHS showed similar results in both conventional training and training with RD (Ekso) (Molteni et al., 2021).

Research using RD (HAL) gait training increased the walking speed and the distance walked during 2-minute walk test (2MWT), cadence, and stride length in subacute stroke (Mizukami et al., 2016). The Berg Balance Scale (BBS), FMA, FAC, and PCI also improved, but not significantly (Mizukami et al., 2016). A randomized clinical trial showed significant improvement in FAC and that the improvement was retained 2 months post-intervention, while maximum walking speed, stride, cadence, 6MWT, TUG, and FMA of the lower extremity did not show an effect (Watanabe et al., 2017). Research using an RD (wearable ankle) showed FAC and walking speed improved after using the RD compared to the control group (Yeung et al., 2021).

###### 3.3.1.1.3. Effects of RD in individuals with chronic stroke

In individuals with chronic stroke, MI, FAC, 10MWT and 6MWT showed improvements, while the Ashworth scale and WHS did not show any improvements after RD (Ekso) training (Molteni et al., 2017). A similar study comparing RD (Ekso) training to the control group also showed minimal clinically important difference (MCID) in 10MWT and 6MWT (Schröder et al., 2019). A randomized study in individuals with chronic stroke showed that the RD (Ekso) group improved in all Coping Orientation to Problems Experienced outcomes, FIM, 10MWT, TUG, and River Mobility Index (RMI), as well as achieved a greater improvement in constipation and QoL than SOC (De Luca et al., 2020). A pilot study compared the wearable RD (Ekso) with both end effector training and SOC. The results showed no significant difference between the three groups (Pournajaf et al., 2019). Although statistical significance was not obtained, pre-post differences in the RD and end effector groups in TUG, 10MWT, and 6MWT were higher than the MCID values reported in the literature. These differences were not found in the SOC group (Pournajaf et al., 2019).

All patients who received HAL training showed significant improvements in 10MWT, cadence, number of steps, TUG, functional reach (FRT), and BBS compared to SOC in chronic stroke patients (Yoshimoto et al., 2015). Similarly, a longitudinal study by Tanaka et al. showed that gait speed, stride length, cadence, and 2MWT were significantly increased after the RD (HAL) gait training, and the effects were retained after 3 months in chronic stroke patients (Tanaka et al., 2019). Similarly, a case study on a participant with chronic stroke showed improvement in 10MWT, TUG, FRT, two-step test, and BBS after RD (HAL) training, and the improvements were retained at 2 month follow-up (Yoshimoto et al., 2016). RD (HAL) intervention significantly improved gait speed, cadence, BBS, and the number of steps assessed by the 10MWT in individuals with chronic stroke. The TUG also improved though was not statistically significant (Kawamoto et al., 2013). The individuals were further divided into independent ambulatory and dependent ambulatory subgroups. Both groups showed significant change in BBS while only the dependent ambulatory subgroup showed statistically significant differences in walking speed, cadence, and number of steps (Kawamoto et al., 2013). Preliminary study on RD (Indego) with participants with chronic TBI and stroke showed improvements in speed and endurance in 10MWT and 6MWT respectively. Some of the participants also improved their FAC level (Jyräkoski et al., 2021).

Preliminary investigation of the RD (H2) gait training in the three chronic stroke patients showed that the participants performed a more symmetric gait in RD training and there were slight improvements in 6MWT, TUG, and FMA-LE in all the participants after training (Bortole et al., 2015). Researchers investigated the effectiveness of the developed RD (Robot-assisted ankle-foot-orthosis) on chronic stroke patients (Yeung et al., 2018). The results showed improvement in FAC and 10MWT while wearing RD while FMA also improved from pre to post-training. There were no significant differences found in MAS, BBS, and 6MWT (Yeung et al., 2018). Walking with passive RD (Exoband) showed that walking distance and gait speed increased in people with stroke after training compared to baseline (Panizzolo et al., 2021). Rehabilitation using ExoAtlet RD in chronic stroke showed that the speed, BBS, and TS showed improvement after RD training (Kovalenko et al., 2021).

##### 3.3.1.2. Biomechanical and physiological outcomes

Research has found a median perceived exertion on the Borg Scale after RD training (Ekso), with a statistically significant change in walking time, standing time, and number of steps with the progression of gait training and MAS in sub-acute stroke patients (Høyer et al., 2020). This study shows somewhat above fairly light exertion training with RD. The study also showed that participants walked greater distances and achieved more steps throughout the training sessions, but with lessened cardiorespiratory strain during RD training. The authors suggest that RD-assisted gait training is less energy consuming and less cardiorespiratory stressful than walking without RD-assistance, encouraging more repetitions in participants. This, in turn, could strengthen motor learning and control (Høyer et al., 2020).

Analysis of the load distribution on feet with open and closed eyes in both groups [RD (Ekso GT) and SOC] showed a small and non-significant tendency to reduce the amount of uninvolved limb loading after therapy, which may indicate gradual improvement in limb loading symmetry (Rojek et al., 2020). Results also indicate that after training with the RD, load distribution between the limbs was better than in SOC (Rojek et al., 2020), though both groups improved their loading characteristics after training. In addition, observed changes in BI and RMI also showed a significant improvement in the RD group compared to the control group (Rojek et al., 2020).

A preliminary study showed that one session of RD (Vanderbilt) training in individuals with stroke resulted in improvements in gait speed, spatial symmetry, and step length (Murray et al., 2014, 2015). The effect of the RD (SMAS) on the biomechanical metrics were evaluated (Buesing et al., 2015). The results showed a significantly large increase in step length and spatial symmetry in the SMAS group than the control group. There was an increase in velocity, stride length and step length on both impaired and non-impaired sides with an associated decrease in swing time on the affected side and double support time for both groups (Buesing et al., 2015).

Researchers quantified improvement of lateral symmetry after using single-leg RD (HAL) using EMG in acute stroke patients (Tan et al., 2018). The results showed a significant increase in similarity between lateral synergies of patients with a corresponding increase in gait measures like walking speed, step length, step cadence, stance duration, and percentage of gait cycle (Tan et al., 2018). In addition, improvements in FIM-locomotion, FIM-motor, and FMA scores were also observed (Tan et al., 2018). Researchers utilized muscle synergy analysis to show gait symmetry in subacute stroke patients that underwent RD (HAL) gait training (Tan et al., 2020). The results showed no significant differences in muscle synergy symmetry between RD and SOC groups though the timing of muscle synergies was symmetrical in the HAL group but not in the control group. Intergroup comparisons of symmetry in muscle synergies and their timings were not significantly different. This could be due to large variability in recovery in the control group. Finally, stance time ratio was not observed to improve in both groups after their respective therapies (Tan et al., 2020). There was a significant increase in FIM-locomotion, FIM-motor, and FMA scores in both the HAL group and control group (Tan et al., 2020).

Muscle strength changes after use of RD (UG0210) using the manual muscle strength test of the tibialis anterior muscle in acute and subacute stroke patients were evaluated. No significant difference was observed between control and RD groups but both groups improved after training (Zhang et al., 2020).

The preliminary study evaluated the effect of RD (Ekso) training on EMG and functional gait in individuals with subacute stroke (Infarinato et al., 2021). MI-affected limb, FAC showed significant improvement, while MAS-Affected Limb, TCT, and 10MWT did not show a change after training (Infarinato et al., 2021). Bilateral Symmetry and mean root mean square improved in TA and co-contraction decreased in proximal muscles after RD training (Infarinato et al., 2021).

Increases in strength in the paretic muscles were noted, along with increases in stability, functional level, and walking speed in the group with RD (ExoAtlet) compared to without RD (Kotov et al., 2021). Comparison of stabilometric also showed improved outcomes in RD compared to without RD (Kotov et al., 2021). An increase in stride length and decrease in gait speed was observed after RD training in acute stroke (Kotov et al., 2021). The effect of RD (Ekso) on neuromuscular coordination was evaluated in individuals with chronic stroke and compared to healthy controls (Zhu et al., 2021). Spatial-temporal parameters, kinematics, and muscle synergy pattern were analyzed. The results showed the motor modules for steadfast walking were described by four distinct motor modules described in heathy controls and three modules in the paretic leg of the stroke patients. Muscle coordination complexity, module composition, and activation timing were preserved after the training. In contrast, walking with the RD altered the stroke subjects' synergy pattern, especially on the paretic side. The changes were dominated by the activation profile modulation toward the normal pattern observed in the healthy controls (Zhu et al., 2021).

Researchers determined whether the newly developed hip-assist robot (GEMS) was effective in improving biomechanical and physiological outcomes in stroke patients (Lee et al., 2019). Gait speed, cadence, stride length, gait symmetry, sEMG of bilateral RF, BF, TA, and MG, cardiopulmonary metabolic energy efficiency, FMA, fall efficacy scale, and BBS were measured. The results showed an improvement in all biomechanical outcomes, except gait symmetry in the RD group. There was an improvement in all muscle activity in RD and only in RF for CGT. There was a decrease in metabolic energy, FMA, and Fall Efficacy Scale for RD (Lee et al., 2019).

Robotic leg orthosis (RLO) is an actuated RD that provided assistance for extension and flexion. Preliminary data showed improved clinical (BBS and LE-FMA) outcomes (Li et al., 2015). The participants also improved their cadence, step length, and walking speed. The EMG results showed there was an increase in the normalized root mean square values of the MG and BF on the affected side. Additionally, EMG activities of the agonist and antagonist pair (i.e., RF and BF) appeared to return to similar levels after training. The peak moment of hip flexor, knee extensor, and plantar flexor, which all contributed to push-off power, were found to have increased after 3 weeks of training (Li et al., 2015).

##### 3.3.1.3. Neurological outcomes

RD (Ekso) training, in hemiplegic chronic stroke patients, resulted in greater improvement with medium to large effect size in 10MW, RMI, TUG, CSE and sensory-motor integration (SMI) using Transcranial Magnetic Stimulation (TMS), frontoparietal effective connectivity (FPEC) using EEG, than SOC (Calabrò et al., 2018). These changes were accompanied by improved stance/swing ratio, gait cycle duration, reduced limb asymmetries, and hip and knee muscle activation. The strengthening of FPEC, the increase of SMI in the affected side, and the decrease of SMI in the unaffected side were the most important factors correlated with the clinical improvement. The RD induced a reshape of CSE of both hemispheres, whereas SOC change mainly pertained to the affected CSE. This research concluded that RD induced a more evident remodulation of SMI between the hemispheres as compared to SOC (Calabrò et al., 2018).

EEG was used to understand short-term changes due to RD (Ekso) training in people with chronic stroke. Study findings showed a strong relation between lesion lateralization, dominance and connectivity modulations (Molteni et al., 2020). Right-hemisphere (non-dominant) stroke participants showed an increase of connectivity Node strength (NS) over the contralesional motor cortex and ipsilesional prefrontal cortex after exoskeleton training. They displayed a modification of connectivity after RD-assisted gait toward a pattern similar to one described during ankle dorsiflexion in able-bodied persons suggesting short term neuroplasticity. In left-hemisphere stroke participants (dominant hemisphere lesion), the connectivity pattern is different. Even after RD-assisted gait, both connectivity betweenness centrality (BC) and NS point to a preeminent role of the vertex node, that is, the scalp field projection of the neurons discharging in the lower limb area. In these persons, a foot task does not activate a complex network, but only a focal activity over the corresponding motor cortex, thereby suggesting a lower degree of local efficiency and reorganization (Molteni et al., 2020).

Research by Jayaraman et al. showed that significant functional improvement in walking speed, berg balance scale, and endurance with an associated change in corticomotor excitability (CME) using TMS after intervention with the RD compared to the control group (Jayaraman et al., 2019). This suggests that RD may promote greater walking speed, endurance, balance, and CME than control.

#### 3.3.2. Soft exoskeletons

**Figure 9:**
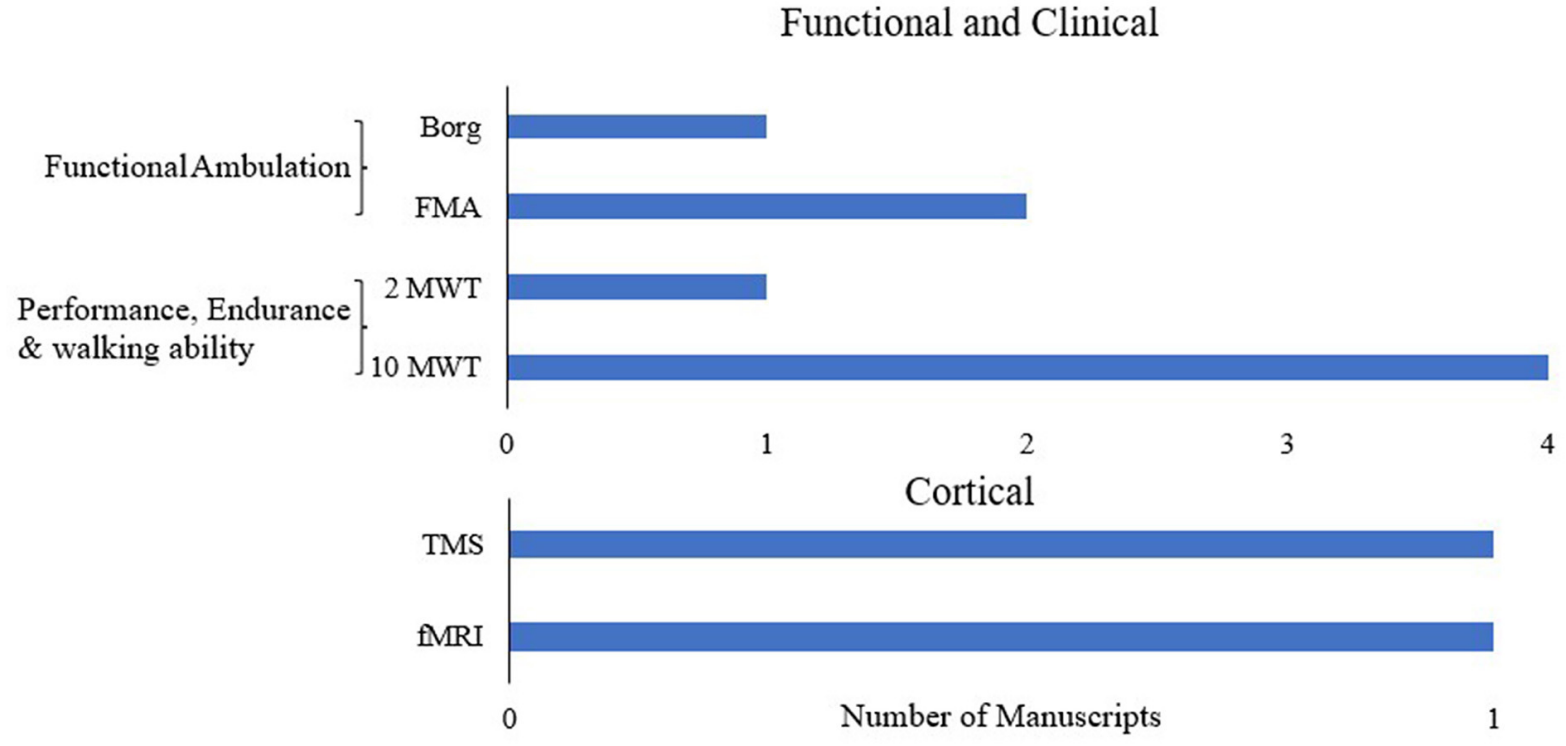
Assessments used across all studies in stroke with soft RD.

Figure 9 shows the distribution of functional and clinical and cortical assessments across all studies in the stroke population. In the limited number of studies utilizing soft RD in the stroke population, the most widely used outcomes are performance, endurance and walking ability measures (10MWT, 2MWT).

##### 3.3.2.1. Functional and clinical outcomes

The feasibility study on RD (ReWalk Restore) showed that individuals with stroke increased their walking speed with and without RD after 5 days of RD training (Awad et al., 2020). It is noteworthy that 36% of study participants achieved a large meaningful increase (i.e., ≥ 0.10 m/s) in their unassisted maximum walking speed after only 5 days of training (Awad et al., 2020). Preliminary feasibility study on RD (Myosuit) showed improvements in walking speed while the distance in 2MWT reduced after four sessions of RD training (Haufe et al., 2020).

Randomized clinical trial in people with subacute stroke showed the RD (Regent) significantly improved gait speed and BBS. In addition, improvements were also observed in FIM and BI (Monticone et al., 2013).

##### 3.3.2.2. Neurological outcomes

Cortical reorganization in 14 patients with chronic stroke after the use of Regent RD was analyzed using fMRI using a passive sensorimotor paradigm imitating the support loading during slow walking (Saenko et al., 2016). The results showed that the temporal characteristics of walking improved, which was accompanied by a decrease in the activation zones of the inferior parietal lobules, especially in the healthy hemisphere, and a significant increase in the activation zones of the primary sensorimotor and supplementary motor areas (Saenko et al., 2016). Functional connectivity analysis showed significant changes in intrahemispheric and interhemispheric interactions (Saenko et al., 2016).

Navigated TMS (nTMS) was used to assess the changes in gait cortical control after using RD (Regent) for 10 sessions in individuals with chronic stroke (Poydasheva et al., 2016). Results showed increased speed with no change in FMA. During nTMS, a reduction was recorded in the average latency of evoked motor response from the affected hemisphere, as well as various patterns of changes in the size and localization of cortical representations of the leg muscles (Poydasheva et al., 2016).

## 4. Discussion

Current research is focused on improving gait and balance mechanisms using RDs for rehabilitation (Federici et al., 2016; Louie and Eng, 2016; Lefeber et al., 2017; Molteni et al., 2018; Moucheboeuf et al., 2020). These robotic devices may provide support for gait and/or balance, assist with joint movements, or reduce the use of compensatory mechanisms during walking. Most of the reviews on RD research have focused on design and development activities in terms of electromechanical design or software controllers with a view to provide optimal control (Dollar and Herr, 2008; Viteckova et al., 2013; Shi et al., 2019; Lee et al., 2020). Many reviews were on gait trainers/non-overground robotic devices, which are very different from overground robotic RDs (Moucheboeuf et al., 2020). Other reviews had a narrower focus; such as reviews on only randomized clinical trials or reviews of safety, ease of use, or feasibility of use in clinical environments (Mehrholz and Pohl, 2012; Poli et al., 2013; Federici et al., 2015, 2016; Schwartz and Meiner, 2015; Wall et al., 2015; Louie and Eng, 2016; Alias et al., 2017; Hill et al., 2017; Jayaraman et al., 2017; Lefeber et al., 2017; Bruni et al., 2018; Mehrholz et al., 2018; Molteni et al., 2018; Weber and Stein, 2018; Postol et al., 2019; Moucheboeuf et al., 2020; Pinto-Fernandez et al., 2020; Swank et al., 2020a; Dijkers et al., 2021; Sale et al., 2021). Though they provide a great insight into the usage of the device, they do not help us to understand the relationship between training, neuroplasticity, and functional recovery. In this paper, we have reviewed various preliminary and pre-clinical, as well as clinical, research that evaluated RDs using functional, clinical, biomechanical, physiological, and cortical outcomes. We carefully delineate between the preliminary studies and RCTs in interpretation of existing evidence. The goal was to provide a review of the state of the science and to provide directions for further investigation. Another feature of this review is that for the first time the downstream (functional, biomechanical and physiological) and upstream (cortical) metrics used to evaluate various RDs in ABI are presented. In order to understand both device effectiveness and neuroplasticity, it is important to understand RD effectiveness in the context of all these metrics. This paper provides a comprehensive review of the clinical and pre-clinical research on the therapeutic (pre-post) effects of various over-ground gait training RDs. This review lays the foundation to understand the knowledge gaps that currently exists in RD rehabilitation research. Though there is initial preliminary evidence on efficacy of RD, a comprehensive randomized controlled clinical trial is required to fully understand the therapeutic effects of RD in ABI.

Our review shows that RDs could be used for gait therapy across age groups, based on the ability to fit into exoskeletons. It has also shown that RDs could be used across deficit characteristics (i.e., diplegia, hemiplegia, and quadriplegia). The selection of an RD would be dependent on the amount of support and control the user requires from the robot. A non-ambulatory participant with ABI would require maximal assistance with rigid supports to walk and balance. On the other hand, an ambulatory participant may require assistance to perform accurate symmetrical gait with limited supports (soft RD), allowing for multidimensional walking and balance. Further research is required to precisely determine the optimal use of rigid and soft RDs at different stages of recovery to allow for treatment progression.

A healthy walking control mechanism involves supporting the body weight and providing stability in the forward and lateral directions during forward progression (Winter, 2009; Perry and Burnfield, 2010). In each step, stability and balance are associated with progression (Winter, 2009; Perry and Burnfield, 2010). The control mechanism needs to be able to scan the environment for obstacles and adjust the gait pattern and balance in order to adapt to the environment. Individuals with ABI often present with loss of voluntary movement, loss of movement co-ordination, muscle weakness, etc., resulting in gait and balance control mechanism deficits (Kerrigan et al., 2000; Williams et al., 2009; Winter, 2009; Kemu, 2010; Perry and Burnfield, 2010; Dubin, 2014; Sheffler and Chae, 2015). Compensatory mechanisms like steppage gait, hip hiking, toe walking, crouch gait, etc. are used to ambulate successfully (Kerrigan et al., 2000; Williams et al., 2009; Winter, 2009; Kemu, 2010; Perry and Burnfield, 2010; Dubin, 2014; Sheffler and Chae, 2015). These pathological deviations from healthy walking result in slower walking speed, shorter step length, reduced gait and balance adaptability, and increased risk of falls (Kerrigan et al., 2000; Williams et al., 2009; Winter, 2009; Kemu, 2010; Perry and Burnfield, 2010; Dubin, 2014; Sheffler and Chae, 2015).

The last decade has seen an enormous growth in the development of RDs for neuro-rehabilitation (Dollar and Herr, 2008; Yan et al., 2015; Esquenazi et al., 2017; Iandolo et al., 2019). Various software (variable assistance, resistance) and mechanical (soft and rigid structures with different actuator mechanisms) designs for RDs have enabled the provision of individual assistance, from supporting a particular segment or joint (multiple degrees of freedom) to complete control and stability to perform gait and balance (Dollar and Herr, 2008; Yan et al., 2015; Esquenazi et al., 2017; Iandolo et al., 2019). There is increasing evidence showing the feasibility and safety of various RDs in ABI (Dollar and Herr, 2008; Yan et al., 2015; Esquenazi et al., 2017; Iandolo et al., 2019). Therapies with RDs are combining intensive therapy with quality gait and balance training (Federici et al., 2016; Louie and Eng, 2016; Lefeber et al., 2017; Molteni et al., 2018; Moucheboeuf et al., 2020).

Research has shown preliminary evidence for the efficacy of using RDs for CP rehabilitation (Bayón et al., 2016, 2018; Lerner et al., 2017a,b; Bulea et al., 2018; Ueno et al., 2019; Fang et al., 2020; Kuroda et al., 2020). RDs in CP have been designed to target either deficit or function. Both kinds of RDs have shown moderate improvement in functional outcomes (such as speed and endurance, Figure 4) and biomechanical outcomes (such as spatial-temporal characteristics, joint angles, and EMG activation, Figure 4) in preliminary studies. Though this is promising, rigorous randomized controlled trials with large sample size on the use of RDs for CP rehabilitation are largely missing to date.

Research on the usage of RDs in TBI (Figure 5) has been scarce. Research is mostly on providing initial evidence on biomechanical characteristics with associated cortical changes after use of RDs in TBI for restoration of gait and balance (Nolan et al., 2018; Karunakaran et al., 2019, 2020a,b). Pre-clinical and randomized clinical trials are lacking for RD use in individuals with TBI, making it hard to assess the efficacy of RDs in TBI patients.

Recently, there has been an increase in pre-clinical and clinical research to understand the functional and clinical changes after RD use in both acute and chronic stages of stroke. The most commonly studied exoskeletons were the Ekso and the HAL (Nilsson et al., 2014; Mizukami et al., 2016; Molteni et al., 2017, 2021; Watanabe et al., 2017; Goffredo et al., 2019; Nolan et al., 2020; Swank et al., 2020b; Taki et al., 2020; Karunakaran et al., 2021; Yeung et al., 2021). Multiple studies have shown that RD increased the walking distance and walking speed during acute and sub-acute stroke rehabilitation (Molteni et al., 2017, 2021; Watanabe et al., 2017; Goffredo et al., 2019; Karunakaran et al., 2021). The results from studies using Ekso RD have shown improvements in Stroke Rehabilitation Assessment of Movement (Swank et al., 2020b), with one of the studies showing significant improvement in FIM (Nolan et al., 2020), while the other did not (Swank et al., 2020b). Results from HAL RD in acute stroke rehabilitation have shown improvements in speed FAC, FIM, and BRs (Nilsson et al., 2014; Taki et al., 2020). A study using BEAR-H1 RD also showed improvement in functional metrics such as speed, endurance and FMA-LA (Li et al., 2021). Multiple studies have shown that Ekso and HAL improved speed, endurance, balance, and functional ambulatory metrics, but not in spasticity index, though not all studies showed significant differences between the control group and the RD gait training group (Nilsson et al., 2014; Mizukami et al., 2016; Molteni et al., 2017, 2021; Watanabe et al., 2017; Goffredo et al., 2019; Nolan et al., 2020; Swank et al., 2020b; Taki et al., 2020; Karunakaran et al., 2021; Yeung et al., 2021). These mixed results in the metric could be due to various characteristics such as dosing (number of steps) differences, the number of RD sessions (Figure 6A), low sample size (Figure 6B) or the differences in therapy focus (deficit focused, intensity focused or joint focused, etc.) gait training (Partridge et al., 2000; Peurala et al., 2009; Hornby et al., 2016). In order to understand the differences in results, randomized control trials with large sample sizes with these characteristics as covariates needs to be conducted. Current studies suggest that utilization of these exoskeletons may increase functional outcomes during the acute and sub-acute stages of recovery when used over multiple sessions. Though Ekso and HAL have very different control mechanisms, both exoskeletons provide bilateral support and assistance to perform gait in the sagittal plane. Further research is required to understand the effect of different control mechanisms on recovery in these individual devices, as well as between these devices. The effect of “number of steps” and “number of sessions” required to induce significant change also needs to be studied in order to better understand the effects and efficacy of each RD.

Similar results have also been observed in chronic stroke. Various RDs such as Ekso, HAL, Ankle RD's, ExoAtlet, ReWalk Restore, Myosuit, and H2 have shown improvement in speed, endurance, and balance; though not all metrics were significant across all RDs (Kawamoto et al., 2013; Bortole et al., 2015; Yoshimoto et al., 2015, 2016; Molteni et al., 2017; Yeung et al., 2018; Pournajaf et al., 2019; Schröder et al., 2019; Tanaka et al., 2019; Awad et al., 2020; De Luca et al., 2020; Haufe et al., 2020; Jyräkoski et al., 2021; Kovalenko et al., 2021; Panizzolo et al., 2021). The results do suggest that repeated consistent practice provided by RDs has the potential to induce recovery even during the chronic phase of stroke, where spontaneous plasticity reduces and recovery trajectory is slow. Even though the assistance provided to the joints, control mechanism, as well as support provided by the RDs were different, each of the RDs showed changes in recovery of function. Stroke can present with deficits across multiple joints. Each of the RDs may target different deficits. It would be beneficial to understand how the different control mechanisms compare at targeting particular deficits to induce recovery and also to understand if it is beneficial to target single or multi-joints based on the deficits.

Physiological outcomes using RD have been measured through self-report exertion (Borg), heart rate, and measures of oxygen consumption. In people with sub-acute stroke, the perceived exertion was above fairly light exertion based on self-reported measures (Høyer et al., 2020). One of the studies reported less energy expenditure with RD (Ekso) training (Mehrholz et al., 2018). The intensity may be varied by varying the RD control settings, resulting in increased user effort. Future studies need to evaluate the effect of RD control settings on user effort and exertion. During the sub-acute stages of recovery, where the user might need maximum assistance, RDs might need to provide more assistance across the joints to facilitate the user to perform higher quality or correct gait. This in turn might reduce the perceived exertion, while allowing the user to perform more consistent and accurate repetitive steps. In addition, exertion, and cardiorespiratory load depend on various other factors such as level of assistance, various robotic control mechanisms, and type of robot. Current research is sparse on analyzing the exertion and cardio-respiratory strain due to RD training in both acute and chronic stroke. Future research is needed to address this shortcoming. Analyzing the cardio-respiratory strain at various assistance levels across different RDs is necessary to truly understand the effect of RD assistance on intensity (exertion and cardio-respiratory load).

Research on multiple RDs from initial studies suggests improved step length, gait symmetry, and gait kinematic after RD use (Monticone et al., 2013; Murray et al., 2014, 2015; Buesing et al., 2015). Similarly, loading characteristics and loading symmetry improved with RD training (Rojek et al., 2020); though it has also been observed that a single leg version of an RD resulted in improved step length with reduced symmetry. Though randomized controlled trials are needed to verify these results.

Preliminary research on EMG activity has shown improved muscle activity and muscle synergies with improved biomechanical and functional parameters in full-scale RDs; though significant differences were observed across studies (Li et al., 2015; Tan et al., 2018, 2020; Lee et al., 2019; Zhang et al., 2020; Infarinato et al., 2021; Kotov et al., 2021; Zhu et al., 2021). Though initial evidence shows improved biomechanical characteristics with RD use, there is still a need to understand RD impact on different biomechanical metrics, such as spatial-temporal characteristics, loading characteristics, EMG, and kinematics. The impact of the number of joint actuations, actuation assistance, as well as the difference between the actuation of both legs and single leg, needs to be studied in order to completely understand the effects of RDs on biomechanical characteristics. In addition, the effect of RDs on the different compensatory mechanisms needs to be studied. What is more, and warrants further study, is the fact that each person might have varying deficits at varying joints and may require customized therapy. This may require assistance or correction at various joints at varying levels. Hence, it is important to not only understand the effect of RDs on the population or on broad measures of ABI, but also their effects with regard to deficit levels. This will help us develop better rehabilitation RDs by customizing interventions.

Initial research on upstream mechanisms showed that CSE, CME, SMI, frontoparietal effective connectivity (FPEC) was better after RD training than conventional gait training in the preliminary analysis (Calabrò et al., 2018; Jayaraman et al., 2019). RD induced a more evident remodulation of SMI between the hemispheres as compared to conventional training. Node strength (NS) over the contralesional motor cortex and ipsilesional prefrontal cortex after exoskeleton training improved and displayed a modification of connectivity after RD assisted gait (Molteni et al., 2020). Preliminary analysis shows that RD is effective in inducing positive cortical re-organization and improved CSE (Poydasheva et al., 2016; Saenko et al., 2016; Calabrò et al., 2018; Jayaraman et al., 2019; Molteni et al., 2020). Understanding the relationship between lesion, and its effects on cortical activity changes as well as recovery due to RD and its effects on cortical activity changes and evaluating its relationship to biomechanical characteristics will help us optimize rehabilitation.

### 4.1. Future directions

The mechanical and software (control) capabilities of each RD are widespread and still evolving with the technology. RDs can be classified as deficit-targeted or function-targeted devices. Deficit-targeted devices focus on a single joint, while function-targeted devices target gait and balance mechanisms. Both kinds of RDs have shown improvement in ambulation, but the comparative effectiveness of such devices needs to be more comprehensively evaluated. It is unclear whether it is more beneficial to use single joint targeted devices or to target gait and balance function, both overall and/or under which circumstances. In addition, the control strategies vary between RDs. Thus, the training could be altered to provide varying levels of assistance (complete to no assistance) or resistance or trajectory guidance. The effect of these varying control strategies on the different stages of recovery also need to be analyzed. In addition, more comprehensive analysis of the length of training, the amount of training (number of sessions, number of steps), intensity, the effect of assistance, and how and when to reduce assistance and how this affects recovery is needed in order to guide the future trajectory of ABI rehabilitation. In order to answer such questions, comprehensive evaluations of various devices need to be compiled, and their effects on downstream and upstream metrics need to be evaluated.

Research has not addressed the effect of age, time since injury, or patient level of injury on recovery after using RDs. Since recovery plateaus during the chronic stages of ABI, it is important to understand the effects of time since injury. Neurological recovery might be influenced by age, especially with younger individuals having different sequelae influenced by their development; resulting in different patterns of recovery. The severity and location of injury (cortical or sub-cortical, localized vs. diffused, various ROIs) might influence the trajectory of recovery. Injury characteristics might influence the functional connectivity related to gait and balance mechanisms, resulting in varying biomechanical and physiological deficits and recovery. All these factors warrant research in their own right and are a necessary prerequisite for a more comprehensive understanding of post-ABI recovery.

RD research is still evolving. We have provided foundation of the preliminary evidence for over ground robotic devices in order to build more rigorously controlled research study as well as RCTs. The review also lays out the systematic framework to evaluate these devices based on diagnosis, domain (functional, biomechanical and neurological) and based on stage of recovery. We need to first evaluate the RDs for each pathology in each domain separately throughout the different stages of recovery. This will help us determine responders and non-responders in various diseases, domain, and recovery phases. This will help us narrow the devices for translation into clinical practice.

## Author contributions

SP, CB, and EL assisted with sorting the manuscript based on the inclusion/exclusion criteria. KK validated the selected articles based on the inclusion/exclusion criteria and drafted the manuscript. KN and SS reviewed the manuscript. All authors contributed to the article and approved the submitted version.
